# Supplementary material for: The Impact of OsERF34 on Rice Grain-Processing Traits and Appearance Quality
Source: Plants (Basel). 2025 May 27;14(11):1633. doi: 10.3390/plants14111633 (PMC12157257; doi:10.3390/plants14111633)
Supplement: Supplementary file 1 [file plants-14-01633-s001.zip › plants-3606234-supplementary.pdf]

Supplementary Table S1. Compliance rate of key quality trait indices for different sample types in 2023.

Category	Rice Type	Samples Tested(n)	Compliance Rate(%)					
			Head Rice Rate %	Chalkiness Degree(%)	Transparency	Alkali Spreading Value	Gel Consistency	Amylose Content
Application	Indica Glutinous	581	61.45	96.90	98.45	94.32	98.80	94.66
	Japonica Glutinous	275	70.18	94.18	83.64	98.18	98.55	73.09
Regional Trial	Indica Glutinous	5919	76.03	96.15	96.57	91.45	99.36	89.22
	Japonica Glutinous	958	77.35	87.47	93.84	90.08	98.33	87.89
Breeding	Indica Glutinous	880	56.48	91.14	87.50	85.91	96.59	80.45
	Japonica Glutinous	546	58.97	95.42	77.29	89.56	98.53	63.92

Supplementary Table S2. Compliance rate of key quality trait indices for different sample types in 2024.

Category	Rice Type	Samples Tested (n)	Compliance Rate (%)					
			Head Rice Rate%	Chalkiness Degree (%)	Transparency	Alkali Spreading Value	Gel Consistency	Amylose Content
Application	Indica Glutinous	712	58.01	92.84	93.54	96.63	97.47	87.08
	Japonica Glutinous	465	66.45	84.95	76.13	95.91	98.71	65.59
Regional Trial	Indica Glutinous	5525	81.27	93.50	94.90	90.26	99.11	88.72
	Japonica Glutinous	640	45.31	78.44	85.47	78.13	99.69	94.69
Breeding	Indica Glutinous	2064	62.21	84.16	88.81	88.61	94.09	77.37
	Japonica Glutinous	1104	69.47	79.08	80.43	91.39	94.47	81.61

Supplementary Table S3. Fertilization practice (kg/hm^2).

Fertilizer Practice	Basal Fertilizer			Tiller-Promoting Fertilizer	Tiller Maintenance Fertilizer	Panicle Fertilizer	
**Fertilizer types**	Urea	DAP	KCl	Urea	Urea	Urea	KCl
**Fertilizer amount**	150	150	112.5	150	75	90	75

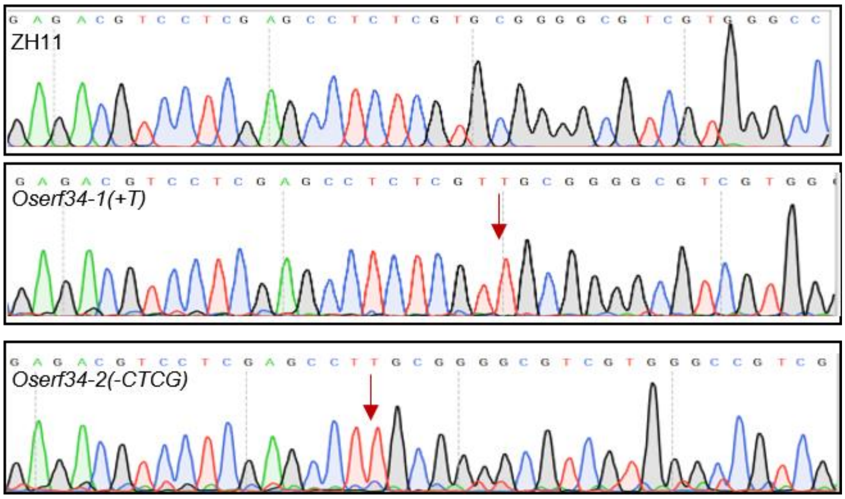

Supplementary Figure S1. CRISPR/Cas9-mediated target mutagenesis of *OsERF34*. Sequence chromatograms of ZH11 (**up**) and *OsERF34* mutant (**down**) at Os04g0550200 locus.

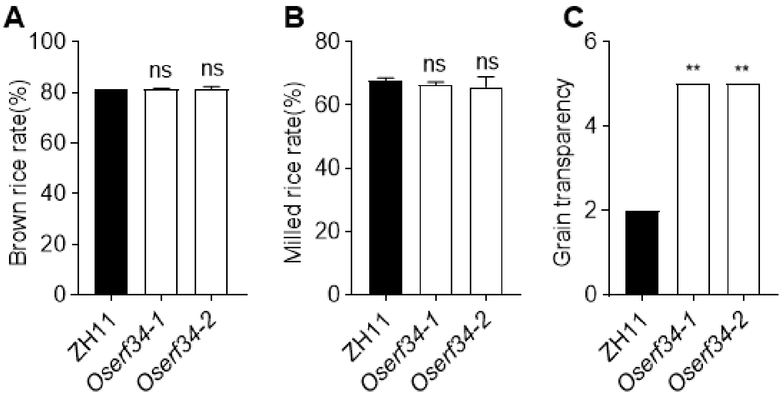

Supplementary Figure S2. Comparison of processing quality between *OsERF34* and ZH11. (**A**–**C**) The brown rice rate, milled rice rate, and grain transparency of ZH11 and *OsERF34*. Error bars represent SD (n = 3). The asterisk and “ns” indicate a significant difference (Student’s *t*-test, $p < 0.01$) and no significant difference between *OsERF34* and ZH11, respectively.

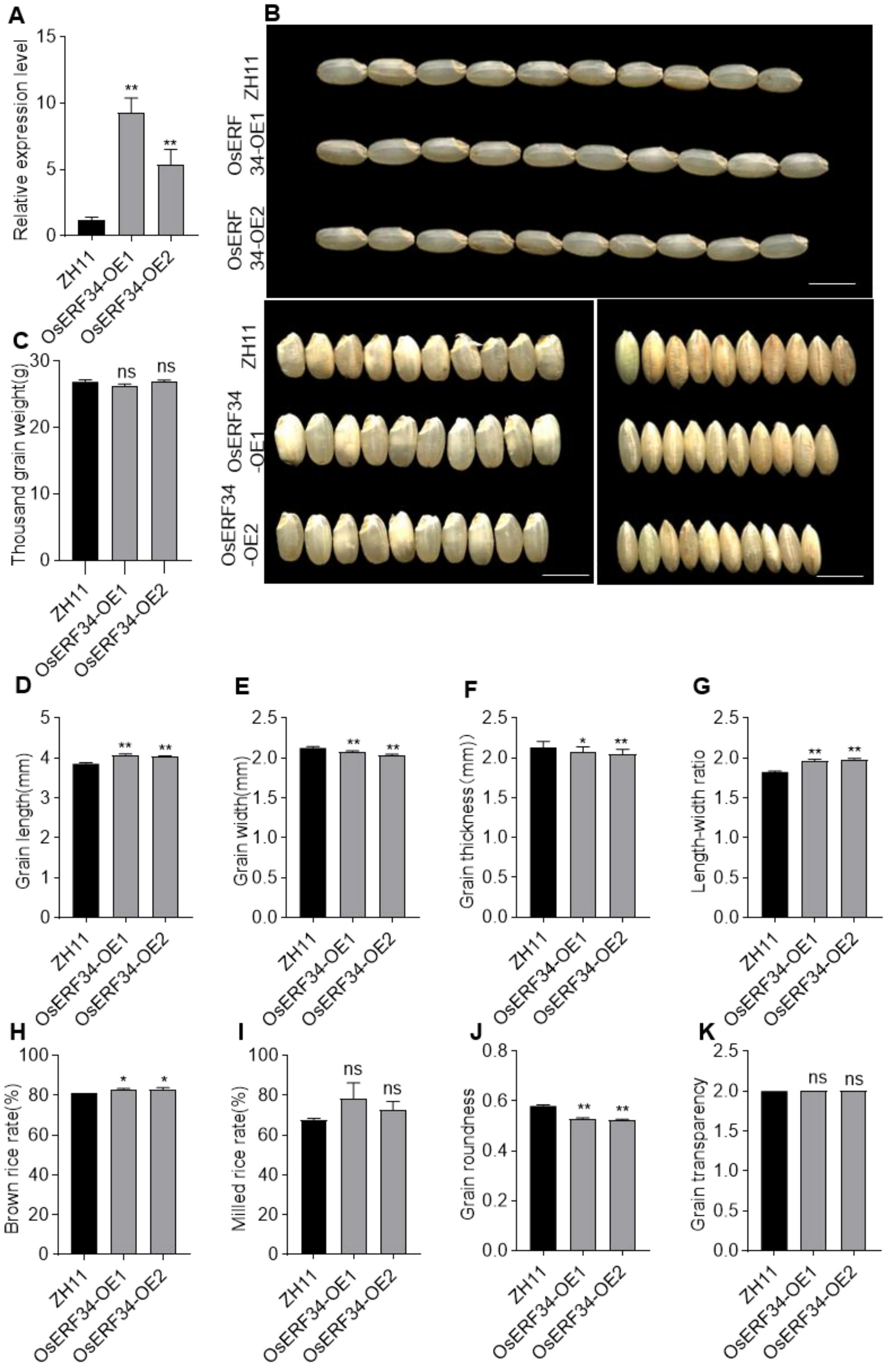

Supplementary Figure S3. Analysis results of ZH11 and OsERF34-OE on rice appearance. (**A**) Relative expression level of *OsERF34* in leaves of ZH11 and OsERF34-OE by quantitative real-time PCR (RT-qPCR). The expression values were normalized to the *Ubq* expression, and wild-type ZH11 was set to 1.0. (**B**–**G**) the phenotypic images ((**B**), scale line: 5 mm) and statistical charts of grain length (**D**), grain width (**E**), grain thickness (**F**), and the length-to-width ratio (**G**) for ZH11 and OsERF34-OE, respectively, (**D**–**F**) were measured using vernier calipers, with 10 randomly selected seeds per measurement and three replicates per sample. (**C**) The thousand-grain weight of ZH11 and OsERF34-OE. (**H**–**J**) The brown rice rate (**H**), milled rice rate (**I**), and grain transparency (**J**) of ZH11 and OsERF34-OE. Error bars represent SD (n = 3), asterisks indicate statistically

notable differences (* $p < 0.05$ and ** $p < 0.01$, Student's t test), and ns indicates no statistically significant difference.

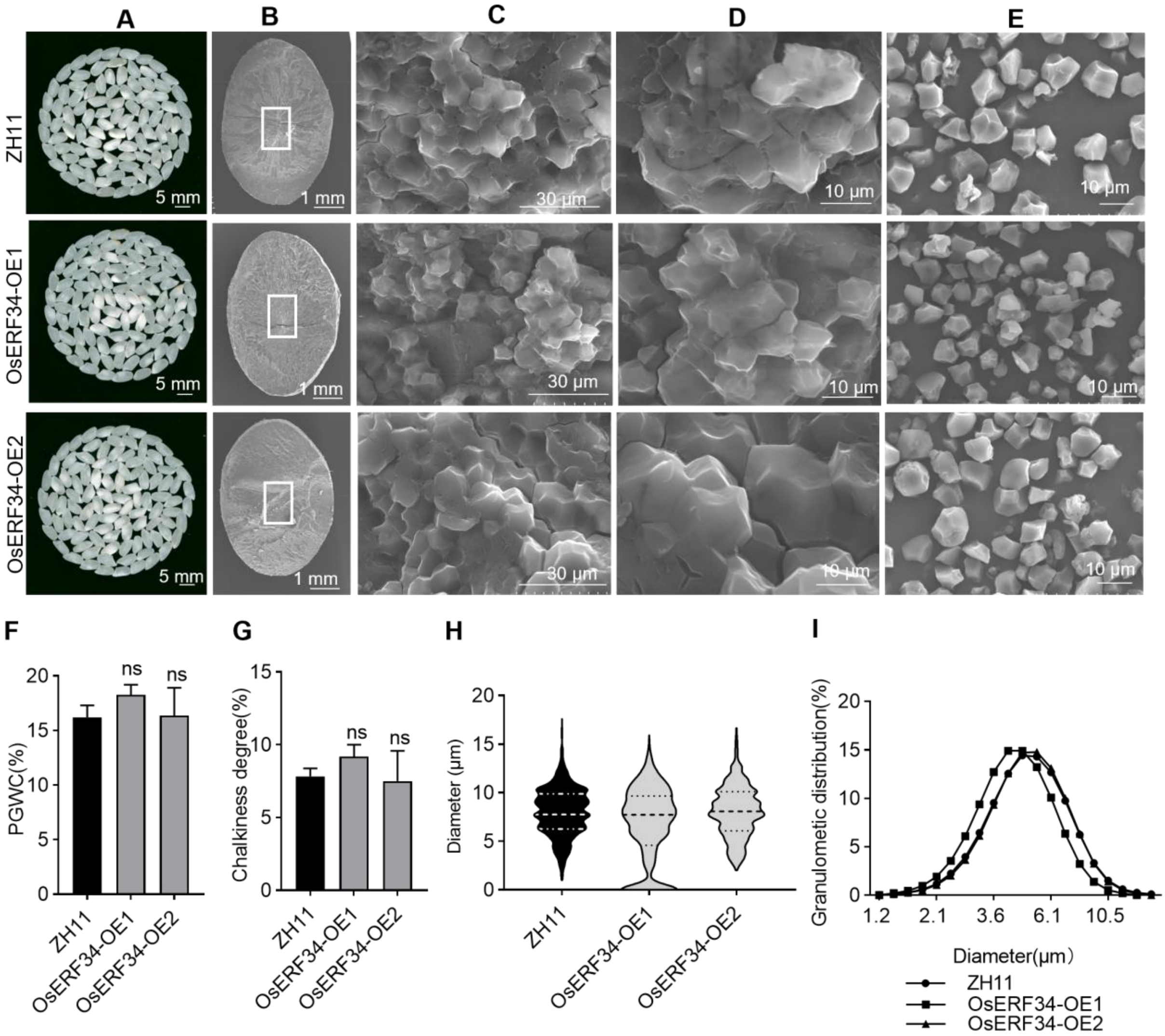

Supplementary Figure S4. Comparison of chalkiness phenotype and starch granules between ZH11 and OsERF34-OE. (**A**) Chalkiness images of ZH11 and OsERF34-OE. (**B**–**D**) Scanning electron microscopy of cross-sections of endosperm in ZH11 and OsERF34-OE, with scale bars of 1 mm (**B**), 30 μm (**C**), and 10 μm (**D**), respectively. The locations indicated by white rectangles correspond to the view positions of images (**C**, **D**, and **E**), Scanning electron microscopy of starch granules in ZH11 and OsERF34-OE. (**F**,**G**), Percentage of grains with chalkiness (PGWC) and chalkiness degree of ZH11 and OsERF34-OE. Error bars represent SD (n = 3). Values are means ± SD (n = 3), asterisks indicate statistically notable differences (* $p < 0.05$ and ** $p < 0.01$, Student's t test), and ns indicates no statistically significant difference. (**H**,**I**), Distribution of starch granule sizes and diameters in ZH11 and OsERF34-OE.

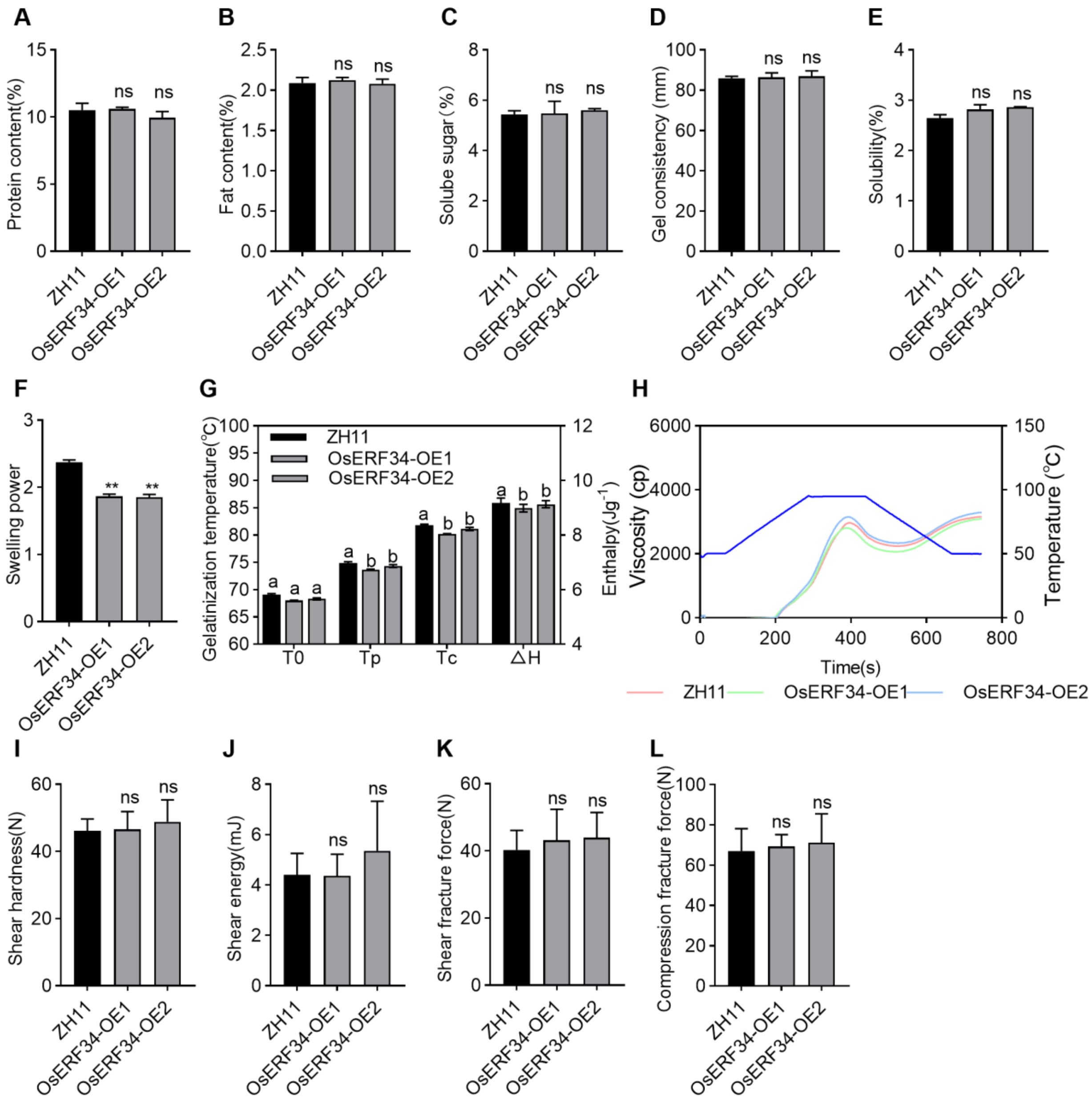

Supplementary Figure S5. The physicochemical properties of the endosperm and seed hardness analysis in mature seeds of OsERF34-OE and ZH11. (**A**–**F**) The protein content (**A**), fat content (**B**), soluble sugar (**C**), gel consistency (**D**), solubility (**E**), and swelling power (**F**) of ZH11 and OsERF34-OE. values are means ± SD (n = 3), asterisks indicate statistically notable differences (* $p < 0.05$ and ** $p < 0.01$, Student's *t* test), and ns indicates no statistically significant difference. (**G**) Differential Scanning Calorimetry (DSC) analysis of milled rice flour of ZH11 and OsERF34-OE. (**H**) Rapid Visco analyzer (RVA) viscosity analysis of ZH11 and OsERF34-OE. (**I**–**L**) Shear hardness (**I**), shear energy (**J**), shear fracture force (**K**), and compression fracture force of mature grains in ZH11 and OsERF34-OE. Error bars represent SD (n = 10), asterisks indicate statistically notable differences (* $p < 0.05$ and ** $p < 0.01$, Student's *t* test), and ns indicates no statistically significant difference.

CDS
>LOC_Os04g46440.1

ATGGACGACTCCCACGACCTGGCCTCCCCGACCTCCCCTGACACGGCGTCCTCGTCGTCT
TCGTCTACGTCGACATCATCGTCCTCCGCCACCGTCGCCCCGAAGAAGCGGCCGCGCAAC
GACGGCCGGCACCCGACGTACCGCGGCGTGCGCATGCGGAGCTGGGGGAAGTGGGTGTCC
GAGATCAGGGAGCCCCGCAAGAAGTCGCGCATCTGGCTGGGCACGTTCGCCACCGCGGAG
ATGGCCGCGCGCGCGCACGACGTGGCCGCGCTCGCCATCAAGGGCCGCACCGCGCACCTC
AACTTCCCCGGACCTCGCGCACCTGCTCCCGCGCCCGGCCACCGCGGCGCCCAAGGACGTG
CAGGCGGCGGCGCTGCTCGCCGCCGCCGCAGCCGACTTCCCCTCCGTCTCCGTCGACGCC
AATGCCAAGAGCCCCGACACCTGCTCCGTCGCCAGCGCCGCCTCGCCGCAGCCGCCACCG
CCGGACGCCGAAGCGGACCCTGACAGCACGCTGTTCGACCTCCCGGACCTGCTCCTGGAC
CTGAGATACGAGACGTCCTCGAGCCTCTCGTGCGGGGCGTCGTGGGCCGTCGATGACGAC
GTGGCCGGCGGCGTCGTGTTCCGCCTCGAGGAGCCCATGCTGTGGGATTACTGA

Supplementary Figure S6. CDS sequence of *OsERF34*.

pCRISPR-Cas9:

GGCCGTCGACGACGACGCGTCCGCCTGCCTGCTTTTGGCCGGCGGGCCGGGCCGCGGTTGTCCGCCGGGTCGGACCGGTCCCTTGGTCGACCGATGCCTCGGCCACGACGATGCTGGGCCCGAAGCGGTAC
GCGAACCCGTGCCGGGCTGCGGCGGATCGCGGCAGCAGGAGCTCCCCGCCCGCCACGGCTGGCTGGATGGTCGCCTCACGCGCCCGCAGGGGTGACGGCGTGGGTGCGGCGGTCAGCCCCGTGATCGGGC
GTCAGACGGCCGCTGAGGGACCGCGACCACCCCTCCCTTCCCGCCTGCCGTCTTTCACCGCCCGGCCGGTCCGCAGACGCCGTACGGCCAGGCCACGGAGGATTACGGGCAGATGGCGCCCACCCGGCACC
CGGGCTGGGGCACCCTGGACAGCACGAAGGCCCGCCCCACTCACCGGGGCGGGCCTTCGCGTTGAGCTAGAGTTCCCGCCAGCCTCGCAGAGCAGGATTCCCGTTGAGCACCGCCAGGTGCGAATAAGGGA
CAGTGAAGAAGGAACACCCGCTCGCGGGTGGGCCTACTTCACCTATCCTGCCCGGCTGACGCCGTTGGATACACCAAGGAAAGTCTACACGAACCCTTTGGCAAAATCCTGTATATCGTGCGAAAAAGGATG
GATATACCGAAAAAATCGCTATAATGACCCCGAAGCAGGGTTATGCAGCGGAAAATGCAGCTCACGGTAACTGATGCCGTATTTGCAGTACCAGCGTACGGCCCACAGAATGATGTCACGCTGAAAATGCCG
GCCTTTGAATGGGTTCATGTGCAGCTCCATCAGCAAAAGGGGATGATAAGTTTATCACCACCGACTATTTGCAACAGTGCCGTTGATCGTGCTATGATCGACTGATGTCATCAGCGGTGGAGTGCAATGTCGTG
CAATACGAATGGCGAAAAGCCGAGCTCATCGGTCAGCTTCTCAACCTTGGGGTTACCCCCGGCGGTGTGCTGCTGGTCCACAGCTCCTTCCGTAGCGTCCGGCCCCTCGAAGATGGGCCACTTGGACTGATCG
AGGCCCTGCGTGCTGCGCTGGGTCCGGGAGGGACGCTCGTCATGCCCTCGTGGTCAGGTCTGGACGACGAGCCGTTCGATCCTGCCACGTCGCCCGTTACACCGGACCTTGGAGTTGTCTCTGACACATTCTG
GCGCCTGCCAAATGTAAAGCGCAGCGCCCATCCATTTGCCTTTGCGGCAGCGGGGCCACAGGCAGAGCAGATCATCTCTGATCCATTGCCCCTGCCACCTCACTCGCCTGCAAGCCCGGTCGCCCGTGTCCAT
GAACTCGATGGGCAGGTACTTCTCCTCGGCGTGGGACACGATGCCAACACGACGCTGCATCTTGCCGAGTTGATGGCAAAGGTTCCCTATGGGGTGCCGAGACACTGCACCATTCTTCAGGATGGCAAGTTGG
TACGCGTCGATTATCTCGAGAATGACCACTGCTGTGAGCGCTTTGCCTTGGCGGACAGGTGGCTCAAGGAGAAGAGCCTTCAGAAGGAAGGTCCAGTCGGTCATGCCTTTGCTCGGTTGATCCGCTCCCGCGA
CATTGTGGCGACAGCCCTGGGTCAACTGGGCCGAGATCCGTTGATCTTCCTGCATCCGCCAGAGGCGGGATGCGAAGAATGCGATGCCGCTCGCCAGTCGATTGGCTGAGCTCATAAGTTCCTATTCCGAAGT
TCCTATTCGAAATGACCGACCAAGCGACGCCCAACCTGCCATCACGAGATTTCGATTCCACCGCCGCCTTCTATGAAAGGTTGGGCTTCGGAATCGTTTTCCGGGACGCCGGCTGGATGATCCTCCAGCGCGG
GGATCTCATGCTGGAGTTCTTCGCCCACCCCAAAAGGATCTAGGTGAAGATCCTTTTTGATAATCTCATGACCAAAATCCCTTAACGTGAGTTTTCGTTCCACTGAGCGTCAGACCCCGTAGAAAAGATCAAAG
GATCTTCTTGAGATCCTTTTTTTCTGCGCGTAATCTGCTGCTTGCAAACAAAAAAACCACCGCTACCAGCGGTGGTTTGTTTGCCGGATCAAGAGCTACCAACTCTTTTTCCGAAGGTAACTGGCTTCAGCAGAG
CGCAGATACCAAATACTGTCCTTCTAGTGTAGCCGTAGTTAGGCCACCACTTCAAGAACTCTGTAGCACCGCCTACATACCTCGCTCTGCTAATCCTGTTACCAGTGGCTGCTGCCAGTGGCGATAAGTCGTGTC
TTACCGGGTTGGACTCAAGACGATAGTTACCGGATAAGGCGCAGCGGTCGGGCTGAACGGGGGGTTCGTGCACACAGCCCAGCTTGGAGCGAACGACCTACACCGAACTGAGATACCTACAGCGTGAGCATT
GAGAAAGCGCCACGCTTCCCGAAGGGAGAAAGGCGGACAGGTATCCGGTAAGCGGCAGGGTCGGAACAGGAGAGCGCACGAGGGAGCTTCCAGGGGGAAACGCCTGGTATCTTTATAGTCCTGTCGGGTTT
CGCCACCTCTGACTTGAGCGTCGATTTTTGTGATGCTCGTCAGGGGGGCGGAGCCTATGGAAAAACGCCAGCAACGCGGCCTTTTTACGGTTCCTGGCCTTTTGCTGGCCTTTTGCTCACATGTTCTTTCCTGCGT
TATCCCCTGATTCTGTGGATAACCGTATTACCGCCTTTGAGTGAGCTGATACCGCTCGCCGCAGCCGAACGACCGAGCGCAGCGAGTCAGTGAGCGAGGAAGCGGAAGAGCGCCCAATACGCAAACCGCCTC
TCCCCGCGCGTTGGCCGATTCATTAATGCAGCTGGCACGACAGGTTTCCCGACTGGAAAGCGGGCAGTGAGCGCAACGCAATTAATGTGAGTTAGCTCACTCATTAGGCACCCCAGGCTTTACACTTTATGCTT
CCGGCTCGTATGTTGTGTGGAATTGTGAGCGGATAACAATTTCACACAGGAAACAGCTATGACCATGATTACGAATTGTACGCGGTCGATCTTGACGGCTGGCGAGAGGTGCGGGGAGGATCTGACCGACGCG
GTCCACACGTGGCACCGCGATGCTGTTGTGGGCACAATCGTGCCGGTTGGTAGGATCGACGGCCATGGGTTTTAGAGCTAGAAATAGCAAGTTAAAATAAGGCTAGTCCGTTATCAACTTGAAAAAGTGGCAC
CGAGTCGGTGCTTTTTTTACGTAGGCGTCAGCTACGATGTTCCGGGGACTGCTGATCCGGTCAGCAGGTGGAAGAGGGACTGGATTCCAAAGTTCTCAATGCTGCTTGCTGTTCTTGAATGGGGGGTCGTTGAC
GACGACATGGCTCGATTGGCGCGACAAGTTGCTGCGATTCTCACCAATAAAAAACGCCCGGCGGCAACCGAGCGTTCTGAACAAATCCAGATGGAGTTCTGAGGTCATTACTGGACCGGATCGGGGATCTGG
GCTGAGGGAGCCGACGGCACGCGGCGGCTCACGGCGTGGCACGCGGAACGTCCGGGCTTGCACCTCACGTCACGTGAGGAGGCAGCGTGGACGGCGTCAGAGAAGGGAGCGGACATATGGACAAGAAGTA
CTCCATCGGCCTCGACATCGGCACCAACTCCGTGGGCTGGGCGGTCATCACCGACGAGTACAAGGTCCCCTCCAAGAAGTTCAAGGTCCTGGGCAACACCGACCGGCACTCGATCAAGAAGAACCTGATCGG
CGCCCTGCTCTTCGACAGCGGCGAGACCGCCGAGGCGACCCGCCTGAAGCGGACCGCGCGTCGCCGCTACACCCGGCGCAAGAACCGCATCTGCTACCTGCAGGAAATCTTCTCCAACGAGATGGCCAAGG
TGGACGACTCGTTCTTCCACCGCCTGGAGGAGAGCTTCCTGGTGGAGGAGGACAAGAAGCACGAGCGCCACCCGATCTTCGGCAACATCGTGGACGAGGTGGCCTACCACGAGAAGTACCCCACCATCTACC
ACCTCCGCAAGAAGCTGGTGGACTCGACCGACAAGGCGGACCTGCGGCTCATCTACCTGGCCCTCGCGCACATGATCAAGTTCCGCGGCCACTTCCTCATCGAGGGCGACCTGAACCCGGACAACTCCGACG
TGGACAAGCTCTTCATCCAGCTGGTGCAGACCTACAACCAGCTGTTCGAGGAGAACCCCATCAACGCCAGCGGCGTGGACGCCAAGGCGATCCTCTCCGCGCGCCTGAGCAAGTCCCGGCGCCTGGAGAAC
CTCATCGCCCAGCTGCCGGGCGAGAAGAAGAACGGCCTCTTCGGCAACCTGATCGCGCTGTCGCTCGGCCTGACCCCCAACTTCAAGAGCAACTTCGACCTGGCCGAGGACGCGAAGCTCCAGCTGTCCAAG
GACACCTACGACGACGACCTGGACAACCTGCTCGCCCAGATCGGCGACCAGTACGCGGACCTCTTCCTGGCCGCGAAGAACCTCTCGGACGCCATCCTGCTCAGCGACATCCTGCGGGTCAACACCGAGATC
ACCAAGGCCCCGCTGTCGGCGAGCATGATCAAGCGGTACGACGAGCACCACCAGGACCTGACCCTGCTCAAGGCCCTCGTGCGCCAGCAGCTGCCCGAGAAGTACAAGGAAATCTTCTTCGACCAGTCCAA
GAACGGCTACGCCGGCTACATCGACGGCGGCGCGTCGCAGGAGGAGTTCTACAAGTTCATCAAGCCGATCCTGGAGAAGATGGACGGCACCGAGGAGCTGCTCGTCAAGCTGAACCGCGAGGACCTGCTCC
GCAAGCAGCGGACCTTCGACAACGGCTCCATCCCGCACCAGATCCACCTGGGCGAGCTCCACGCCATCCTCCGGCGCCAGGAGGACTTCTACCCCTTCCTGAAGGACAACCGCGAGAAGATCGAGAAGATC
CTGACCTTCCGCATCCCGTACTACGTCGGCCCCCTGGCCCGCGGCAACTCCCGGTTCGCGTGGATGACCCGGAAGTCGGAGGAGACCATCACCCCGTGGAACTTCGAGGAGGTCGTGGACAAGGGCGCGTCC
GCGCAGTCGTTCATCGAGCGCATGACCAACTTCGACAAGAACCTCCCGAACGAGAAGGTCCTGCCCAAGCACTCCCTGCTCTACGAGTACTTCACCGTGTACAACGAGCTGACCAAGGTCAAGTACGTGACC
GAGGGCATGCGGAAGCCGGCCTTCCTGTCGGGCGAGCAGAAGAAGGCGATCGTGGACCTGCTCTTCAAGACCAACCGCAAGGTCACCGTGAAGCAGCTGAAGGAGGACTACTTCAAGAAGATCGAGTGCTT
CGACTCCGTCGAGATCAGCGGCGTGGAGGACCGCTTCAACGCCTCCCTGGGCACCTACCACGACCTGCTCAAGATCATCAAGGACAAGGACTTCCTCGACAACGAGGAGAACGAGGACATCCTGGAGGACA
TCGTCCTCACCCTGACCCTCTTCGAGGACCGCGAGATGATCGAGGAGCGGCTCAAGACCTACGCCCACCTGTTCGACGACAAGGTGATGAAGCAGCTGAAGCGTCGCCGCTACACCGGCTGGGGCCGCCTCT
CCCGGAAGCTGATCAACGGCATCCGGGACAAGCAGAGCGGCAAGACCATCCTGGACTTCCTCAAGTCCGACGGCTTCGCCAACCGCAACTTCATGCAGCTCATCCACGACGACAGCCTGACCTTCAAGGAGG
ACATCCAGAAGGCCCAGGTCTCGGGCCAGGGCGACAGCCTCCACGAGCACATCGCCAACCTGGCGGGCTCCCCGGCGATCAAGAAGGGCATCCTCCAGACCGTCAAGGTCGTGGACGAGCTGGTCAAGGTG
ATGGGCCGCCACAAGCCCGAGAACATCGTGATCGAGATGGCCCGGGAGAACCAGACCACCCAGAAGGGCCAGAAGAACTCGCGCGAGCGGATGAAGCGGATCGAGGAGGGCATCAAGGAGCTCGGCAGC
CAGATCCTGAAGGAGCACCCGGTCGAGAACACCCAGCTGCAGAACGAGAAGCTGTACCTCTACTACCTGCAGAACGGCCGCGACATGTACGTGGACCAGGAGCTCGACATCAACCGGCTGTCCGACTACGA
CGTGGACCACATCGTGCCGCAGTCCTTCCTGAAGGACGACTCGATCGACAACAAGGTCCTGACCCGCTCGGACAAGAACCGGGGCAAGTCCGACAACGTGCCCTCGGAGGAGGTCGTGAAGAAGATGAAGA
ACTACTGGCGCCAGCTGCTCAACGCCAAGCTCATCACCCAGCGCAAGTTCGACAACCTGACCAAGGCCGAGCGGGGCGGCCTGAGCGAGCTCGACAAGGCGGGCTTCATCAAGCGCCAGCTGGTCGAGACC
CGGCAGATCACCAAGCACGTGGCCCAGATCCTGGACTCCCGGATGAACACCAAGTACGACGAGAACGACAAGCTGATCCGCGAGGTCAAGGTGATCACCCTCAAGAGCAAGCTGGTCTCCGACTTCCGCAA
GGACTTCCAGTTCTACAAGGTCCGGGAGATCAACAACTACCACCACGCCCACGACGCGTACCTGAACGCCGTCGTGGGCACCGCGCTGATCAAGAAGTACCCGAAGCTGGAGTCCGAGTTCGTCTACGGCGA
CTACAAGGTCTACGACGTGCGCAAGATGATCGCCAAGAGCGAGCAGGAGATCGGCAAGGCCACCGCGAAGTACTTCTTCTACTCCAACATCATGAACTTCTTCAAGACCGAGATCACCCTGGCCAACGGCGA
GATCCGCAAGCGGCCCCTGATCGAGACCAACGGCGAGACCGGCGAGATCGTCTGGGACAAGGGCCGCGACTTCGCCACCGTCCGGAAGGTGCTGTCGATGCCGCAGGTCAACATCGTGAAGAAGACCGAG
GTGCAGACCGGCGGCTTCAGCAAGGAGTCCATCCTCCCCAAGCGCAACAGCGACAAGCTGATCGCCCGGAAGAAGGACTGGGACCCGAAGAAGTACGGCGGCTTCGACAGCCCCACCGTCGCCTACTCCGT
GCTGGTCGTGGCGAAGGTCGAGAAGGGCAAGAGCAAGAAGCTGAAGTCCGTGAAGGAGCTGCTCGGCATCACCATCATGGAGCGCTCCTCGTTCGAGAAGAACCCGATCGACTTCCTGGAGGCCAAGGGCT
ACAAGGAGGTCAAGAAGGACCTCATCATCAAGCTGCCCAAGTACAGCCTGTTCGAGCTGGAGAACGGCCGCAAGCGGATGCTCGCCTCCGCGGGCGAGCTGCAGAAGGGCAACGAGCTGGCCCTCCCGTCG
AAGTACGTCAACTTCCTGTACCTCGCGTCCCACTACGAGAAGCTGAAGGGCTCGCCCGAGGACAACGAGCAGAAGCAGCTCTTCGTGGAGCAGCACAAGCACTACCTGGACGAGATCATCGAGCAGATCAG
CGAGTTCAGCAAGCGCGTCATCCTGGCCGACGCGAACCTCGACAAGGTGCTGTCCGCCTACAACAAGCACCGCGACAAGCCGATCCGGGAGCAGGCGGAGAACATCATCCACCTGTTCACCCTCACCAACC
TGGGCGCCCCCGCCGCGTTCAAGTACTTCGACACCACCATCGACCGCAAGCGGTACACCTCCACCAAGGAGGTCCTCGACGCGACCCTGATCCACCAGAGCATCACCGGCCTGTACGAGACCCGCATCGACC
TGTCCCAGCTCGGCGGCGACTGAAAGCTTTCTAGAGGATCCCCGGGTACCGAGCTCGAATTCCCCAGATCTAAAGTTTTGTCGTCTTTCCAGACGTTAGTAAATGAATTTTCTGTATGAGGTTTTGCTAAACAAC
TTTCAACAGTTTCAGCGGAGTGAGAATAGAAAGGAACAACTAAAGGAATTGCGAATAATAATTTTTTCACGTTGAAAATCTCCAAAAAAAAAGGCTCCAAAAGGAGCCTTTAATTGTATCGGTTTATCAGCTTG
CTTTCGAGGTGAATTTCTTAAACAGCTTGATACCGATAGTTGCGCCGACAATGACAACAACCATCGCCCACGCATAACCGATATATTCGGTCGCTGAGGCTTGCAGGGAGTCAAAGGCCGCTTTTGCGGGATCT
CGTCGAAGGCACTAGAAGGCCTATATGCGGGGATCGACCGCGCGGGTCCCGGACGGGGAAGAGCGGGGAGCTTTGCCAGAGAGCGACGACTTCCCCTTGCGTTGGTGATTGCCGGTCAGGGCAGCCATCCG
CCATCGTCGCGTAGGGTGTCACACCCCAGGAATCGCGTCACTGAACACAGCAGCCGGTAGGACGACCATGACTGAGTTGGACACCATCGCAAATCCGTCCGATCCCGCGGTGCAGCGGATCATCGATGTCAC
CAAGCCGTCGCGATCCAACATAAAGACAACGTTGATCGAGGACGTCGAGCCCCTCATGCACAGCATCGCGGCCGGGGTGGAGTTCATCGAGGTCTACGGCAGCGACAGCAGTCCTTTTCCATCTGAGTTGCTG
GATCTGTGCGGGCGGCAGAACATACCGGTCCGCCTCATCGACTCCTCGATCGTCAACCAGTTGTTCAAGGGGGAGCGGAAGGCCAAGACATTCGGCATCGCCCGCGTCCCTCGCCCGGCCAGGTTCGGCGAT
ATCGCGAGCCGGCGTGGGGACGTCGTCGTTCTCGACGGGGTGAAGATCGTCGGGAACATCGGCGCGATAGTACGCACGTCGCTCGCGCTCGGAGCGTCGGGGATCATCCTGGTCGACAGTGACATCACCAGC
ATCGCGGACCGGCGTCTCCAAAGGGCCAGCCGAGGTTACGTCTTCTCCCTTCCCGTCGTTCTCTCCGGTCGCGAGGAGGCCATCGCCTTCATTCGGGACAGCGGTATGCAGCTGATGACGCTCAAGGCGGATG
GCGACATTTCCGTGAAGGAACTCGGGGACAATCCGGATCGGCTGGCCTTGCTGTTCGGCAGCGAAAAGGGTGGGCCTTCCGACCTGTTCGAGGAGGCGTCTTCCGCCTCGGTTTCCATCCCCATGATGAGCCA
GACCGAGTCTCTCAACGTTTCCGTTTCCCTCGGAATCGCGCTGCACGAGAGGATCGACAGGAATCTCGCGGCCAACCGATAAGCGCCTCTGTTCCTCGGACGCTCGGTTCCTCGACCTCGATTCGTCAGTGATG
ATCAACAGGACCCAGAAATGGCACGAGCCCGGGATTGGCCTCCCGGGCTCGCCGCCCCCGAACCGGCGCAGCATGCCGAGTGAGGACGTTTGCTCTGTGACGAGCGTACGAGAGAACAGCGCGTCGACACC
ACCGCGCCCCGCCGGACCGAAGTGCCACTGCGGCACCCCGATCGTCCACATCCCCGGGAAGCGCCCCAAAATCTACTGCTCGGACGCCTGCCGAAAGCGCGCAAAGCGTCTCATTGCGGACCAGTCACGCA
GCGTAGCAAGCACGACTACAGCGGGTAATCCGGACGCGGGCTTTAGTAGTAAGGGAAAATCTCCGTCCACTGCAGGTCAGAGCGCTGTAACTGGAACGGGTTCTACCCCAAACGAGGGTGCCGCGACAGCC
GGGCGGGAGGACCCGCGCGACGAGCGGTTCGCCCGGCGGGACCGGCACCAGACGGTGTCGCTGAACGAGGCGTTCAAGGGCTGCGGGACCCGGCTCACTGCCGGGACGGCCGAGCTGATGTGGAAGCCG
GGCGAGGCGACGTGGGGCAACACGTGCCGCTGCAACAACGTCCACACCTGCCCGTGGTGCATGAGCCGCATCCTGGCCGTACGGGGCTCGAACGTGCAGCTCGCCGCCGACGGTCTCGCGGACGCCGGGTA
CGGGCTGCACCTTGGCACGAACACGTTGCGCCACTTCGAGCGCATGGCGTTCGGCACCGTCCGCAAGGGAATGCGGCACGGCCTGGTCGCGGTCTTGCACGACGGCTGGAAGGGCGCGTACGGCTCGTCCG
GCCGCCGCTGGCGCACGATGCGCGACGACTTCGGGATCATCGGCTACGAGCGGGCCTTCGAGGACACCTTCGGCTGGGGCTCCGGCTGGCACCTGCACTGGCACACGCTCTGGGTCACCCGCGAGGTGCTCG
GCCCGGACGCCCAGGCCGCGTTCCGCGACGCACTCGCCGGAGCCTGGGCCGCCGGGGTCGAATCCGCGGGCGGCTACACCGTCAGCGAGACGTGCGACCGGCCCGGTTGCTCCTGTGAGGGCAAGGGCCA
CGGCACCGACGTGCGCCCACTCAACGGCGCGGACGCCGCTGACGGCGATGCGGGCAAGCAGGCCCGCTACCTCTACAAGGACGGCGACAAGACCAAGGGCGGCGTCGCCAAGATCGGCCTGGAGCTGGCC
GGACAGAACTTCAAGGCCGGTCGCGGCGACGACCGCATGGGACCGCTCGACCTGGGCGACGCGGCGGCCGCCGAGCTGCAGCGGCTTCGTCGGCCCGGCCCGTTCGTCGAGAAGTACCGCGAGCGCGAGT
TCGGCGTCTTCCAGGTCCGCAAGCACTACCGGTCGCAGAACCTGAACCGGCTGATCAAGGAACTCGGCATCCAGCAGGACGTCCGCACCGAGGAGGAGATCACCGACGACACCGAGGGCCTGGTCGCCATC
GCGGTCATCCCGGCCTACATCTGGTACCGATACATCGCCCGCGTCGCCGGACGTCGCCTCGACCTCATCAAGGTCGCCGAGACGTACGGCCTACCGGGCGTCCGGCGGCTCGTCGAGTCCTGGGGCCTGGTG
TGGGGCAAGGACGTCCTGGACCCGCCCGCCCCGGAAGCGCCGGCCGCGCCGGGCGACCTTGACGCTGACCAGATGAGGTTCGAGGTCATGTCCGAGGAGGAAGCTGCTTTCCGCGAGGCGCGCCGGAAGG
CCAACGAGGCCCGCACGGAGGAGCTGGCCGCTTCGCTCGACCGAGTGCGTCAGCCGAAGAAGGAGGCGATCCGCCCGACGATCAGCCTTCGGAAGCGCCTGAAGCCGAAGCCGGTCACCGTCGACGTCAA
GACTCCCCCGCCCGGCGCCGCGTCGCCCGTGTGCCGCCGCTGCAAGGGCAAGCTCGCCCCGGTACTCCAGCCCTGGGGCCGGCTCCCCGGAGACTGCCTCCGCGTCGACACAGCGGTCGCGTAGGGCCGCT
GAGGGACCGCCATGAGACCGCCACCCCGGACATCCGGGTGGCGGTCTCATGGCGGTCCCGGCGCGAAAGCCGAGGGTCTGCCTGCCGTGAGGTGGCCGGCGGACCTGGGTTACTGCTGGAATCCCGCGCCC
AGTCCGAGCGTCCGCCGGCGCTTTTCACCTGGCTCCGGGTGCTCGTCGATGAGCACCA

Supplementary Figure S7. pCRISPR-Cas9 sequence.

PUN-3FLAG

agattacggggggaattccttttcccaccgctcattcacatttcccattcatcggcccgccgtaaataaaatagacaccccctcaaacaccctctttcccccaacctcgtgttgttcggaggcgcacaacacacacaaaccagatctcccccaaaatccacccgtcgg
cacctccgcttcaaggtacgccgctcgtcctcccccccccccctctctaccttctctagatcggcgttccggtccatggttagggcccggtagttctacttctgttcatgtttgtgttagatccgtgtttgtgttagatccgtgctgctagcgttcgtacacggatgcgac
ctgtacgtcagacacgttctgattgctaacttgccagtgtttctctttggggaatcctgggatggctctagccgttccgcagacgggatcgatttcatgattttttttgtttcgttgcatagggtttggtttgcccttttcctttatttcaatatatgccgtgcacttgtttgtcg
ggtcatcttttcatgcttttttttgtcttggttgtgatgatgtggtctggttgggcggtcgttctagatcggagtagaattctgtttcaaactacctggtggatttattaattttggatctgtatgtgtgtgccatacatattcatagttacgaattgaagatgatggatggaa
atatcgatctaggataggtatacatgttgatgcgggttttactgatgcatatacagagatgctttttgttcgcttggttgtgatgatgtggtgtggttgggcggtcgttcattcgttctagatcggagtagaatactgtttcaaactacctggtgtatttattaattttggaa
ctgtatgtgtgtgtcatacatcttcatagttacgagtttaagatggatggaaatatcgatctaggataggtatacatgttgatgtgggttttactgatgcatatacatgatggcatatgcagcatctattcatatgctctaaccttgagtacctatctattataataaacaag
tatgttttataattattttgatcttgatatacttggatgatggcatatgcagcagctatatgtggatttttttagccctgccttcatacgctatttatttgcttggtactgtttcttttgtcgatgctcaccctgttgtttggtgttacttctgcaggtcgactctagaggatccatgg
attacaaggatcatgatggagactacaaggatcatgatattgattacaaagatgatgacgataagggatccccgggtaccgagctcgaatttccccgatcgttcaaacatttggcaataaagtttcttaagattgaatcctgttgccggtcttgcgatgattatcatat
aatttctgttgaattacgttaagcatgtaataattaacatgtaatgcatgacgttatttatgagatgggtttttatgattagagtcccgcaattatacatttaatacgcgatagaaaacaaaatatagcgcgcaaactaggataaattatcgcgcgcggtgtcatctatgtt
actagatcgggaattcgtaatcatgtcatagctgtttcctgtgtgaaattgttatccgctcacaattccacacaacatacgagccggaagcataaagtgtaaagcctggggtgcctaatgagtgagctaactcacattaattgcgttgcgctcactgcccgctttcca
gtcgggaaacctgtcgtgccagctgcattaatgaatcggccaacgcgcggggagaggcggtttgcgtattggctagagcagcttgccaacatggtggagcacgacactctcgtctactccaagaatatcaaagatacagtctcagaagaccaaagggctatt
gagacttttcaacaaagggtaatatcgggaaacctcctcggattccattgcccagctatctgtcacttcatcaaaaggacagtagaaaaggaaggtggcacctacaaatgccatcattgcgataaaggaaaggctatcgttcaagatgcctctgccgacagtgg
tcccaaagatggacccccacccacgaggagcatcgtggaaaaagaagacgttccaaccacgtcttcaaagcaagtggattgatggggaacatgggggagcacgacactctcgtctactccaagaatatcaaagatacagtctcagaagaccaaagggct
attgagacttttcaacaaagggtaatatcgggaaacctcctcggattccattgcccagctatctgtcacttcatcaaaaggacagtagaaaaggaaggtggcacctacaaatgccatcattgcgataaaggaaaggctatcgttcaagatgcctctgccgacagt
ggtcccaaagatggacccccacccacgaggagcatcgtggaaaaagaagacgttccaaccacgtcttcaaagcaagtggattgatgtgatatctccactgacgtaagggatgacgcacaatcccactatccttcgcaagacccttcctctatataaggaagttc
atttcatttggagaggacacgctgaaatcaccagtctctctctacaaatctatctctctcgagctttcgcagatccgggggggcaatgagatatgaaaaagcctgaactcaccgcgacgtctgtcgagaagtttctgatcgaaaagttcgacagcgtctccgacctg
atgcagctctcggagggcgaagaatctcgtgctttcagcttcgatgtaggagggcgtggatatgtcctgcgggtaaatagctgcgccgatggtttctacaaagatcgttatgtttatcggcactttgcatcggccgcgctcccgattccggaagtgcttgacattg
gggagtttagcgagagcctgacctattgcatctcccgccgttcacagggtgtcacgttgcaagacctgcctgaaaccgaactgcccgctgttctacaaccggtcgcggaggctatggatgcgatcgctgcggccgatcttagccagacgagcgggttcggcc
cattcggaccgcaaggaatcggtcaatacactacatggcgtgatttcatatgcgcgattgctgatccccatgtgtatcactggcaaactgtgatggacgacaccgtcagtgcgtccgtcgcgcaggctctcgatgagctgatgctttgggccgaggactgcccc
gaagtccggcacctcgtgcacgcggatttcggctccaacaatgtcctgacggacaatggccgcataacagcggtcattgactggagcgaggcgatgttcggggattcccaatacgaggtcgccaacatcttcttctggaggccgtggttggcttgtatggag
cagcagacgcgctacttcgagcggaggcatccggagcttgcaggatcgccacgactccgggcgtatatgctccgcattggtcttgaccaactctatcagagcttggttgacggcaatttcgatgatgcagcttgggcgcagggtcgatgcgacgcaatcgtc
cgatccggagccgggactgtcgggcgtacacaaatcgcccgcagaagcgcggccgtctggaccgatggctgtgtagaagtactcgccgatagtggaaaccgacgccccagcactcgtccgagggcaaagaatagagtagatgccgaccggatctg
tcgatcgacaagctcgagtttctccataataatgtgtgagtagttcccagataagggaattagggttcctatagggtttcgctcatgtgttgagcatataagaaacccttagtatgtatttgtatttgtaaaatacttctatcaataaaatttctaattcctaaaaccaaaat
ccagtactaaaatccagatcccccgaattaattcggcgttaattcagtacattaaaaacgtccgcaatgtgttattaagttgtctaagcgtcaatttgtttacaccacaatatatcctgccaccagccagccaacagctccccgaccggcagctcggcacaaaatcacc
actcgatacaggcagcccatcagtccgggacggcgtcagcgggagagccgttgtaaggcggcagactttgctcatgttaccgatgctattcggaagaacggcaactaagctgccgggtttgaaacacggatgatctcgcggagggtagcatgttgattgta
acgatgacagagcgttgctgcctgtgatcaccgcggtttcaaaatcggctccgtcgatactatgttatacgccaactttgaaaacaactttgaaaaagctgttttctggtatttaaggttttagaatgcaaggaacagtgaattggagttcgtcttgttataattagcttc
ttggggtatctttaaatactgtagaaaagaggaaggaaataataaatggctaaaatgagaatatcaccggaattgaaaaaactgatcgaaaaataccgctgcgtaaaagatacggaaggaatgtctcctgctaaggtatataagctggtgggagaaaatgaa
aacctatatttaaaaatgacggacagccggtataaagggaccacctatgatgtggaacgggaaaaggacatgatgctatggctggaaggaaagctgcctgttccaaaggtcctgcactttgaacggcatgatggctggagcaatctgctcatgagtgaggc
cgatggcgtcctttgctcggaagagtatgaagatgaacaaagccctgaaaagattatcgagctgtatgcggagtgcatcaggctctttcactccatcgacatatcggattgtccctatacgaatagcttagacagccgcttagccgaattggattacttactgaata
acgatctggccgatgtggattgcgaaaactgggaagaagacactccatttaaagatccgcgcgagctgtatgatttttttaaagacggaaaagcccgaagaggaacttgtcttttcccacggcgacctgggagacagcaacatctttgtgaaagatggcaaagt
aagtggctttattgatcttgggagaagcggcagggcggacaagtggtatgacattgccttctgcgtccggtcgatcaggaggatatcggggaagaacagtatgtcgagctatttttgacttactggggatcaagcctgattgggagaaaataaaatattata
ttttactggatgaattgttttagtacctagaatgcatgaccaaaatcccttaacgctgagagatcccctcataatttccccaaagcgtaaccatgtgtgaataaattttgagctagtagggttgcagccacgagtaagtcttcccttgttattgtgtagccagaatgccg
caaaacttccatgcctaagcgaactgttgagagtacgtttcgatttctgactgtgttagcctggaagtgcttgtcccaaccttgtttctgagcatgaacgcccgcaagccaacatgttagttgaagcatcagggcgattagcagcatgatatcaaaacgctctgagc
tgctcgttcggctatggcgtaggcctagtccgtaggcaggacttttcaagtctcggaaggtttcttcaatctgcattcgcttcgaatagatattaacaagttgtttgggtgttcgaatttcaacaggtaagttagttgctagaatccatggctcctttgccgacgctgag
tagattttaggtgacgggtggtgacaatgagtccgtgtcgagcgctgatttttttcggcctttagagcgagatttatacaatagaatttggcatgagattggattgcttttagtcagcctcttatagcctaaagtctttgagtgactagatgacatatcatgtaagttgct
gataggtttccagttttccgctcctaggtctgcatattgtacttttcctcttactcgacttaaccagtaccaacccagcttctcaacggatttataccatggcactttaaagccagcatcactgacaatgagcggtgtggtgttactcggtagaatgctcgcaaggtcggc
tagaaattggtcatgagctttctttgaacattgctctgaaagcgggaacgctttctcataaagagtaacagaacgaccgtgtagtgcgactgaagctcgcaataccataagccgtttttgctcacggatatcagaccagtcaacaagtacaatgggcatcgtattgc
ccgaacagataaagctagcatgccaacggtatacagcgagtcgctctttgtggaggtgacgattacctaacaatcggtcgattcgtttgatgttatgttttgttctcgctttggttggcaggttacggccaagttcggtaagagtgagagttttacagtcaagtaag
gcgtggcaagccaacgttaagctgttgagtcgttttaagtgtaattcggggcagaattggtaaagagagtcgtgtaaaatatcgagttcgcacattttgttgtctgattattgatttttggcgaaaccatttgatcatatgacaagatgtgtatctaccttaacttaatga
ttttgataaaaaatcattaggggattcatcagcccttaacgtgagttttcgttccactgagcgtcagaccccgtagaaaagatcaaaggatcttcttgagatcctttttttctgcgcgtaatctgctgcttgcaaacaaaaaaaccaccgctaccagcggtggtttgtttgc
cggatcaagagctaccaactctttttccgaaggtaactggcttcagcagagcgcagataccaaatactgtccttctagtgtagccgtagttaggccaccacttcaagaactctgtagcaccgcctacatacctcgctctgctaatcctgttaccagtggctgctgcca
gtggcgataagtcgtgtcttaccgggttggactcaagacgatagttaccggataaggcgcagcggtcgggctgaacggggggttcgtgcacacagcccagcttggagcgaacgacctacaccgaactgagatacctacagcgtgagctatgagaaagcg
ccacgcttcccgaagggagaaaggcggacaggtatccggtaagcggcagggtcggaacaggagagcgcacgagggagcttccagggggaaacgcctggtatctttatagtcctgtcgggtttcgccacctctgacttgagcgtcgatttttgtgatgctcg
tcaggggggcggagcctatggaaaaacgccagcaacgcggcctttttacggttcctggccttttgctggccttttgctcacatgttctttcctgcgttatcccctgattctgtggataaccgtattaccgcctttgagtgagctgataccgctcgccgcagccgaacg
accgagcgcagcgagtcagtgagcgaggaagcggaagagcgcctgatgcggtattttctccttacgctctgtgcggtatttcacaccgcatatggtgcactctcagtacaatctgctctgatgccgcatagttaagccagtatacactccgctatcgctacgtga
ctgggtcatggctgcgccccgacacccgccaacacccgctgacgcgccctgacgggcttgtctgctcccggcatccgcttacagacaagctgtgaccgtctccgggagctgcatgtgtcagaggttttcaccgtcatcaccgaaacgcgcgaggcagggtg
ccttgatgtgggcgccggcggtcgagtggcgacggcgcggcttgtccgcgccctggtagattgcctggccgtaggccagccatttttgagcggccagcggccgcgataggccgacgcgaagcggcggggcgtagggagcgcagcgaccgaagggt
aggcgctttttgcagctcttcggctgtgcgctggccagacagttatgcacaggccaggcgggttttaagagttttaataagttttaaagagttttaggcggaaaaatcgccttttttctcttttatatcagtcacttacatgtgtgaccggttcccaatgtacggctttgg
gttcccaatgtacgggttccggttcccaatgtacggctttgggttcccaatgtacgtgctatccacaggaaagagaccttttcgacctttttcccctgctagggcaatttgccctagcatctgctccgtacattaggaaccggcggatgcttcgccctcgatcaggttg
cggtagcgcatgactaggatcgggccagcctgccccgcctcctccttcaaatcgtactccggcaggtcatttgacccgatcagcttgcgcacggtgaaacagaacttcttgaactctccggcgctgccactgcgttcgtagatcgtcttgaacaaccatctggctt
ctgccttgcctgcggcgcggcgtgccaggcggtagagaaaacggccgatgccgggatcgatcaaaaagtaatcggggtgaaccgtcagcacgtccgggttcttgccttctgtgatctcgcggtacatccaatcagctagctcgatctcgatgtactccggcc
gcccggtttcgctctttacgatcttgtagcggctaatcaaggcttcaccctcggataccgtcaccaggcggccgttcttggccttcttcgtacgctgcatggcaacgtgcgtggtgtttaaccgaatgcaggtttctaccaggtcgtctttctgctttccgccatcggct
cgccggcagaacttgagtacgtccgcaacgtgtggacggaacacgcggccgggcttgtctcccttcccttcccggtatcggttcatggattcggttagatgggaaaccgccatcagtaccaggtcgtaatcccacacactggccatgccggccggccctgcg
gaaacctctacgtgcccgtctggaagctcgtagcggatcacctcgccagctcgtcggtcacgcttcgacagacggaaaacggccacgtccatgatgctgcgactatcgcgggtgcccacgtcatagagcatcggaacgaaaaaatctggttgctcgtcgccc
ttgggcggcttcctaatcgacggcgcaccggctgccggcggttgccgggattctttgcggattcgatcagcggccgcttgccacgattcaccggggcgtgcttctgcctcgatgcgttgccgctgggcggcctgcgcggccttcaacttctccaccaggtcatc
acccagcgccgcgccgatttgtaccgggccggatggtttgcgaccgctcacgccgattcctcgggcttgggggttccagtgccattgcagggccggcaggcaacccagccgcttacgcctggccaaccgcccgttcctccacacatggggcattccacggc
gtcggtgcctggttgttcttgattttccatgccgcctcctttagccgctaaaattcatctactcatttattcatttgctcatttactctggtagctgcgcgatgtattcagatagcagctcggtaatggtcttgccttggcgtaccgcgtacatcttcagcttggtgtgatcctc
cgccggcaactgaaagttgacccgcttcatggctggcgtgtctgccaggctggccaacgttgcagccttgctgctgcgtgcgctcggacggccggcacttagcgtgtttgtgcttttgctcattttctctttacctcattaactcaaatgagttttgatttaatttcagc
ggccagcgcctggacctcgcgggcagcgtcgccctcgggttctgattcaagaacggttgtgccggcggcggcagtgcctgggtagctcacgcgctgcgtgatacgggactcaagaatgggcagctcgtacccggccagcgcctcggcaacctcaccgcc
gatgcgcgtgcctttgatcgcccgcgacacgacaaaggccgcttgtagccttccatccgtgacctcaatgcgctgcttaaccagctccaccaggtcggcggtggcccatatgtcgtaagggcttggctgcaccggaatcagcacgaagtcggctgccttgatc
gcggacacagccaagtccgccgcctggggcgctccgtcgatcactacgaagtcgcgccggccgatggccttcacgtcgcggtcaatcgtcgggcggtcgatgccgacaacggttagcggttgatcttcccgcacggccgcccaatcgcgggcactgccct
ggggatcggaatcgactaacagaacatcggccccggcgagttgcagggcgcgggctagatgggttgcgatggtcgtcttgcctgacccgcctttctggttaagtacagcgataaccttcatgcgttccccttgcgtatttgtttatttactcatcgcatcatatacg
cagcgaccgcatgacgcaagctgttttactcaaatacacatcacctttttagacggcggcgctcggtttcttcagcggccaagctggccggccaggccgccagcttggcatcagacaaaccggccaggatttcatgcagccgcacggttgagacgtgcgcgg
gcggctcgaacacgtacccggccgcgatcatctccgcctcgatctcttcggtaatgaaaaacggttcgtcctggccgtcctggtgcggtttcatgcttgttcctcttggcgttcattctcggcggccgccagggcgtcggcctcggtcaatgcgtcctcacggaag
gcaccgcgccgcctggcctcggtgggcgtcacttcctcgctgcgctcaagtgcgcggtacagggtcgagcgatgcacgccaagcagtgcagccgcctctttcacggtgcggccttcctggtcgatcagctcgcgggcgtgcgcgatctgtgccggggtga
gggtagggcgggggccaaacttcacgcctcgggccttggcggcctcgcgcccgctccgggtgcggtcgatgattagggaacgctcgaactcggcaatgccggcgaacacggtcaacaccatgcggccggccggcgtggtggtgtcggcccacggctc
tgccaggctacgcaggcccgcgccggcctcctggatgcgctcggcaatgtccagtaggtcgcgggtgctgcgggccaggcggtctagcctggtcactgtcacaacgtcgccagggcgtaggtggtcaagcatcctggccagctccgggcggtcgcgcct
ggtgccggtgatcttctcggaaaacagcttggtgcagccggccgcgtgcagttcggcccgttggttggtcaagtcctggtcgtcggtgctgacgcgggcatagcccagcaggccagcggcggcgctcttgttcatggcgtaatgtctccggttctagtcgca
agtattctactttatgcgactaaaacacgcgacaagaaaacgccaggaaaagggcagggcggcagcctgtcgcgtaacttaggacttgtgcgacatgtcgttttcagaagacggctgcactgaacgtcagaagccgactgcactatagcagcggaggggtt
ggatcaaagtactttgatcccgaggggaaccctgtggttggcatgcacatacaaatggacgaacggataaaccttttcacgcccttttaaatatccgattattctaataaacgctcttttctcttaggtttacccgccaatatatcctgtcaaacactgatagtttaattcc
cgatctagtaacatagatgacaccgcgcgcgataatttatcctagtttgcgcgctatattttgttttctatcgcgtattaaatgtataattgcgggactctaatcataaaaacccatctcataaataacgtcatgcattacatgttaattattacatgcttaacgtaattcaaca
gaaattatatgataatcatcgcaagaccggcaacaggattcaatcttaagaaactttattgccaaatgtttgaacgatcggggaaattcgagctggtcacctgtaattcacacgtggtggtggtggtggtggctagcttgtttgcctccctgctgcggtttttcaccg
aagttcatgccagtccagcgtttttgcagcagaaaagccgccgacttcggtttgcggtcgcgagtgaagatccctttcttgttaccgccaacgcgcaatatgccttgcgaggtcgcaaaatcggcgaaattccatacctgttcaccgacgacggcgctgacgcg
atcaaagacgcggtgatacatatccagccatgcacactgatactcttcactccacatgtcggtgtacattgagtgcagcccggctaacgtatccacgccgtattcggtgatgataatcggctgatgcagtttctcctgccaggccagaagttctttttccagtaccttc
tctgccgtttccaaatcgccgctttggacataccatccgtaataacggttcaggcacagcacatcaaagagatcgctgatggtatcggtgtgagcgtcgcagaacattacattgacgcaggtgatcggacgcgtcgggtcgagtttacgcgttgcttccgccagt
ggcgcgaaatattcccgtgcaccttgcggacgggtatccggttcgttggcaatactccacatcaccacgcttgggtgggttttgtcacgcgctatcagctctttaatcgcctgtaagtgcgcttgctgagtttccccgttgactgcctcttcgct

Supplementary Figure S8. PUN-3FLAG sequence.
