# Supplementary material for: The Impact of OsERF34 on Rice Grain-Processing Traits and Appearance Quality
Source: Plants (Basel). 2025 May 27;14(11):1633. doi: 10.3390/plants14111633 (PMC12157257; doi:10.3390/plants14111633)
Supplement: Supplementary file 1 [file Supplementary file 2 for reviewer 2.pdf › Supplementary file 2 for reviewer 2.pdf]

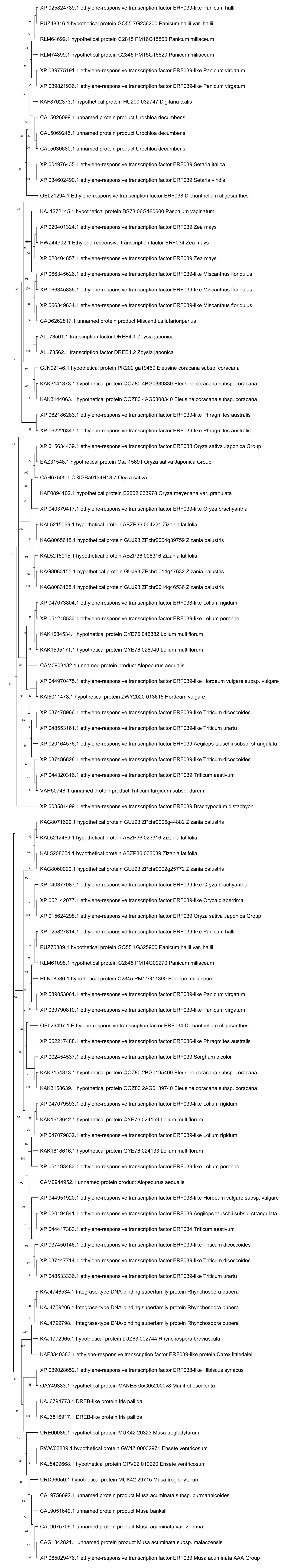
XP 025824789.1 ethylene-responsive transcription factor ERF039-like Panicum hallii
PUZ48316.1 hypothetical protein GQ55 7G236200 Panicum hallii var. hallii
RLM64699.1 hypothetical protein C2845 PM16G15860 Panicum miliaceum
RLM74899.1 hypothetical protein C2845 PM15G16620 Panicum miliaceum
XP 039775191.1 ethylene-responsive transcription factor ERF039-like Panicum virgatum
XP 039821936.1 ethylene-responsive transcription factor ERF039-like Panicum virgatum
KAF8702373.1 hypothetical protein HU200 032747 Digitaria exilis
CAL5026099.1 unnamed protein product Urochloa decumbens
CAL5069245.1 unnamed protein product Urochloa decumbens
CAL5030680.1 unnamed protein product Urochloa decumbens
XP 004976435.1 ethylene-responsive transcription factor ERF039 Setaria italica
XP 034602490.1 ethylene-responsive transcription factor ERF039 Setaria viridis
OEL21294.1 Ethylene-responsive transcription factor ERF038 Dichanthelium oligosanthes
KAJ1272145.1 hypothetical protein BS78 06G180600 Paspalum vaginatum
XP 020401324.1 ethylene-responsive transcription factor ERF039 Zea mays
PWZ44902.1 Ethylene-responsive transcription factor ERF034 Zea mays
XP 020404857.1 ethylene-responsive transcription factor ERF039 Zea mays
XP 066345626.1 ethylene-responsive transcription factor ERF039-like Miscanthus floridulus
XP 066345836.1 ethylene-responsive transcription factor ERF039-like Miscanthus floridulus
XP 066349634.1 ethylene-responsive transcription factor ERF039-like Miscanthus floridulus
CAD6262817.1 unnamed protein product Miscanthus lutarioriparius
ALL73561.1 transcription factor DREB4.1 Zoysia japonica
ALL73562.1 transcription factor DREB4.2 Zoysia japonica
GJN02146.1 hypothetical protein PR202 ga19469 Eleusine coracana subsp. coracana
KAK3141873.1 hypothetical protein QOZ80 4BG0339330 Eleusine coracana subsp. coracana
KAK3144063.1 hypothetical protein QOZ80 4AG0308340 Eleusine coracana subsp. coracana
XP 062186283.1 ethylene-responsive transcription factor ERF039-like Phragmites australis
XP 062226347.1 ethylene-responsive transcription factor ERF039-like Phragmites australis
XP 015634439.1 ethylene-responsive transcription factor ERF038 Oryza sativa Japonica Group
EAZ31548.1 hypothetical protein OsJ 15691 Oryza sativa Japonica Group
CAH67505.1 OSIGBa0134H18.7 Oryza sativa
KAF0894102.1 hypothetical protein E2562 033978 Oryza meyeriana var. granulata
XP 040379417.1 ethylene-responsive transcription factor ERF039-like Oryza brachyantha
KAL5215069.1 hypothetical protein ABZP36 004221 Zizania latifolia
KAG8065618.1 hypothetical protein GUJ93 ZPchr0004g39759 Zizania palustris
KAL5216915.1 hypothetical protein ABZP36 008316 Zizania latifolia
KAG8083155.1 hypothetical protein GUJ93 ZPchr0014g47632 Zizania palustris
KAG8083138.1 hypothetical protein GUJ93 ZPchr0014g46536 Zizania palustris
XP 047073804.1 ethylene-responsive transcription factor ERF038-like Lolium rigidum
XP 051218533.1 ethylene-responsive transcription factor ERF039-like Lolium perenne
KAK1684534.1 hypothetical protein QYE76 045382 Lolium multiflorum
KAK1595171.1 hypothetical protein QYE76 026949 Lolium multiflorum
CAM0903482.1 unnamed protein product Alopecurus aequalis
XP 044970475.1 ethylene-responsive transcription factor ERF039-like Hordeum vulgare subsp. vulgare
KAI5011478.1 hypothetical protein ZWY2020 013615 Hordeum vulgare
XP 037478966.1 ethylene-responsive transcription factor ERF039-like Triticum dicoccoides
XP 048553161.1 ethylene-responsive transcription factor ERF039-like Triticum urartu
XP 020164576.1 ethylene-responsive transcription factor ERF039 Aegilops tauschii subsp. strangulata
XP 037486828.1 ethylene-responsive transcription factor ERF039-like Triticum dicoccoides
XP 044320316.1 ethylene-responsive transcription factor ERF039 Triticum aestivum
VAH50748.1 unnamed protein product Triticum turgidum subsp. durum
XP 003581499.1 ethylene-responsive transcription factor ERF039 Brachypodium distachyon
KAG8071699.1 hypothetical protein GUJ93 ZPchr0006g44882 Zizania palustris
KAL5212469.1 hypothetical protein ABZP36 023316 Zizania latifolia
KAL5208654.1 hypothetical protein ABZP36 033089 Zizania latifolia
KAG8060020.1 hypothetical protein GUJ93 ZPchr0002g25772 Zizania palustris
XP 040377087.1 ethylene-responsive transcription factor ERF039-like Oryza brachyantha
XP 052142077.1 ethylene-responsive transcription factor ERF039-like Oryza glaberrima
XP 015624298.1 ethylene-responsive transcription factor ERF039 Oryza sativa Japonica Group
XP 025827814.1 ethylene-responsive transcription factor ERF039-like Panicum hallii
PUZ76889.1 hypothetical protein GQ55 1G325900 Panicum hallii var. hallii
RLM61098.1 hypothetical protein C2845 PM14G09270 Panicum miliaceum
RLN08536.1 hypothetical protein C2845 PM11G11390 Panicum miliaceum
XP 039853061.1 ethylene-responsive transcription factor ERF039-like Panicum virgatum
XP 039790810.1 ethylene-responsive transcription factor ERF039-like Panicum virgatum
OEL29497.1 Ethylene-responsive transcription factor ERF034 Dichanthelium oligosanthes
XP 062217488.1 ethylene-responsive transcription factor ERF036-like Phragmites australis
XP 002454537.1 ethylene-responsive transcription factor ERF039 Sorghum bicolor
KAK3154813.1 hypothetical protein QOZ80 2BG0195400 Eleusine coracana subsp. coracana
KAK3158639.1 hypothetical protein QOZ80 2AG0139740 Eleusine coracana subsp. coracana
XP 047079593.1 ethylene-responsive transcription factor ERF039-like Lolium rigidum
KAK1618642.1 hypothetical protein QYE76 024159 Lolium multiflorum
XP 047079832.1 ethylene-responsive transcription factor ERF039-like Lolium rigidum
KAK1618616.1 hypothetical protein QYE76 024133 Lolium multiflorum
XP 051193483.1 ethylene-responsive transcription factor ERF039-like Lolium perenne
CAM0944952.1 unnamed protein product Alopecurus aequalis
XP 044951920.1 ethylene-responsive transcription factor ERF038-like Hordeum vulgare subsp. vulgare
XP 020194841.1 ethylene-responsive transcription factor ERF039 Aegilops tauschii subsp. strangulata
XP 044417383.1 ethylene-responsive transcription factor ERF034 Triticum aestivum
XP 037450146.1 ethylene-responsive transcription factor ERF039-like Triticum dicoccoides
XP 037447714.1 ethylene-responsive transcription factor ERF039-like Triticum dicoccoides
XP 048533336.1 ethylene-responsive transcription factor ERF039-like Triticum urartu
KAJ4746534.1 Integrase-type DNA-binding superfamily protein Rhynchospora pubera
KAJ4759206.1 Integrase-type DNA-binding superfamily protein Rhynchospora pubera
KAJ4799798.1 Integrase-type DNA-binding superfamily protein Rhynchospora pubera
KAJ1702965.1 hypothetical protein LUZ63 002744 Rhynchospora breviuscula
KAF3340383.1 ethylene-responsive transcription factor ERF039-like protein Carex littledalei
XP 039028652.1 ethylene-responsive transcription factor ERF038-like Hibiscus syriacus
OAY49383.1 hypothetical protein MANES 05G052000v8 Manihot esculenta
KAJ6794773.1 DREB-like protein Iris pallida
KAJ6816917.1 DREB-like protein Iris pallida
URE00086.1 hypothetical protein MUK42 20323 Musa troglodytarum
RWW03839.1 hypothetical protein GW17 00032971 Ensete ventricosum
KAJ8499668.1 hypothetical protein OPV22 010220 Ensete ventricosum
URD96050.1 hypothetical protein MUK42 29715 Musa troglodytarum
CAL9756692.1 unnamed protein product Musa acuminata subsp. burmannicoides
CAL9051640.1 unnamed protein product Musa banksii
CAL9075706.1 unnamed protein product Musa acuminata var. zebrina
CAG1842821.1 unnamed protein product Musa acuminata subsp. malaccensis
XP 065029476.1 ethylene-responsive transcription factor ERF039 Musa acuminata AAA Group
