# Supplementary material for: The Impact of OsERF34 on Rice Grain-Processing Traits and Appearance Quality
Source: Plants (Basel). 2025 May 27;14(11):1633. doi: 10.3390/plants14111633 (PMC12157257; doi:10.3390/plants14111633)
Supplement: Supplementary file 1 [file Supplementary file 3 for reviewer 2.pdf › Supplementary file 3 for reviewer 2.pdf]

CDS

>LOC_Os04g46440.1

ATGGACGACTCCCACGACCTGGCCTCCCCGACCTCCCCTGACACGGCGTCCTCGTCGTCT
TCGTCTACGTCGACATCATCGTCCTCCGCCACCGTCGCCCCGAAGAAGCGGCCGCGCAAC
GACGGCCGGCACCCGACGTACCGCGGCGTGCGCATGCGGAGCTGGGGGAAGTGGGTGTCC
GAGATCAGGGAGCCCCGCAAGAAGTCGCGCATCTGGCTGGGCACGTTCGCCACCGCGGAG
ATGGCCGCGCGCGCGCACGACGTGGCCGCGCTCGCCATCAAGGGCCGCACCGCGCACCTC
AACTTCCCGGACCTCGCGCACCTGCTCCCGCGCCCGGCCACCGCGGCGCCCAAGGACGTG
CAGGCGGCGGCGCTGCTCGCCGCCGCCGCAGCCGACTTCCCCTCCGTCTCCGTCGACGCC
AATGCCAAGAGCCCCGACACCTGCTCCGTCGCCAGCGCCGCCTCGCCGCAGCCGCCACCG
CCGGACGCCGAAGCGGACCCTGACAGCACGCTGTTCGACCTCCCGGACCTGCTCCTGGAC
CTGAGATACGAGACGTCCTCGAGCCTCTCGTGCGGGGCGTCGTGGGCCGTCGATGACGAC
GTGGCCGGCGGCGTCGTGTTCCGCCTCGAGGAGCCCATGCTGTGGGATTACTGA

pCRISPR-Cas9

GGCCGTCGACGACGACGCGTCCGCCTGCCTGCTTTTGGCCGGCGGGCCGGGCCGCGGTTGTCC
GCCGGGTCGGACCGGTCCCTTGGTCGACCGATGCCTCGGCCACGACGATGCTGGGCCCGAAG
CGGTACGCGAACCCGTGCCGGGCTGCGGCGGATCGCGGCAGCAGGAGCTCCCCGCCCGCCAC
GGCTGGCTGGATGGTCGCCTCACGCGCCCGCAGGGGTGACGGCGTGGGTGCGGCGGTCAGCC
CCGTGATCGGGCGTCAGACGGCCGCTGAGGGACCGCGACCACCCCTCCCTTCCCGCCTGCCGT
CTTTCACCGCCCGGCCGGTCCGCAGACGCCGTACGGCCAGGCCACGGAGGATTACGGGCAGA
TGGCGCCCACCCGGCACCCGGGCTGGGGCACCCTGGACAGCACGAAGGCCCGCCCCACTCAC
CGGGGCGGGCCTTCGCGTTGAGCTAGAGTTCCCGCCAGCCTCGCAGAGCAGGATTCCCGTTGA
GCACCGCCAGGTGCGAATAAGGGACAGTGAAGAAGGAACACCCGCTCGCGGGTGGGCCTACT
TCACCTATCCTGCCCGGCTGACGCCGTTGGATACACCAAGGAAAGTCTACACGAACCCTTTGGC
AAAATCCTGTATATCGTGCGAAAAAGGATGGATATACCGAAAAAATCGCTATAATGACCCCGAA
GCAGGGTTATGCAGCGGAAAATGCAGCTCACGGTAACTGATGCCGTATTTGCAGTACCAGCGT
ACGGCCCACAGAATGATGTCACGCTGAAAATGCCGGCCTTTGAATGGGTTCATGTGCAGCTCCA
TCAGCAAAAGGGGATGATAAGTTTATCACCACCGACTATTTGCAACAGTGCCGTTGATCGTGCT
ATGATCGACTGATGTCATCAGCGGTGGAGTGCAATGTCGTGCAATACGAATGGCGAAAAGCCG
AGCTCATCGGTCAGCTTCTCAACCTTGGGGTTACCCCCGGCGGTGTGCTGCTGGTCCACAGCTC
CTTCCGTAGCGTCCGGCCCCTCGAAGATGGGCCACTTGGACTGATCGAGGCCCTGCGTGCTGC
GCTGGGTCCGGGAGGGACGCTCGTCATGCCCTCGTGGTCAGGTCTGGACGACGAGCCGTTCGA
TCCTGCCACGTCGCCCGTTACACCGGACCTTGGAGTTGTCTCTGACACATTCTGGCGCCTGCCA
AATGTAAAGCGCAGCGCCCATCCATTTGCCTTTGCGGCAGCGGGGCCACAGGCAGAGCAGATC
ATCTCTGATCCATTGCCCCTGCCACCTCACTCGCCTGCAAGCCCGGTCGCCCGTGTCCATGAAC
TCGATGGGCAGGTACTTCTCCTCGGCGTGGGACACGATGCCAACACGACGCTGCATCTTGCCG
AGTTGATGGCAAAGGTTCCCTATGGGGTGCCGAGACACTGCACCATTCTTCAGGATGGCAAGTT
GGTACGCGTCGATTATCTCGAGAATGACCACTGCTGTGAGCGCTTTGCCTTGGCGGACAGGTGG
CTCAAGGAGAAGAGCCTTCAGAAGGAAGGTCCAGTCGGTCATGCCTTTGCTCGGTTGATCCGCT
CCCGCGACATTGTGGCGACAGCCCTGGGTCAACTGGGCCGAGATCCGTTGATCTTCCTGCATCC
GCCAGAGGCGGGATGCGAAGAATGCGATGCCGCTCGCCAGTCGATTGGCTGAGCTCATAAGTT
CCTATTCCGAAGTTCCTATTCGAAATGACCGACCAAGCGACGCCCAACCTGCCATCACGAGATT
TCGATTCCACCGCCGCCTTCTATGAAAGGTTGGGCTTCGGAATCGTTTTCCGGGACGCCGGCTG

GATGATCCTCCAGCGCGGGGATCTCATGCTGGAGTTCTTCGCCCACCCCAAAAGGATCTAGGTG
AAGATCCTTTTTGATAATCTCATGACCAAAATCCCTTAACGTGAGTTTTCGTTCCACTGAGCGTCA
GACCCCGTAGAAAAGATCAAAGGATCTTCTTGAGATCCTTTTTTTCTGCGCGTAATCTGCTGCTT
GCAAACAAAAAAACCACCGCTACCAGCGGTGGTTTGTTTGCCGGATCAAGAGCTACCAACTCTT
TTTCCGAAGGTAACTGGCTTCAGCAGAGCGCAGATACCAAATACTGTCCTTCTAGTGTAGCCGT
AGTTAGGCCACCACTTCAAGAACTCTGTAGCACCGCCTACATACCTCGCTCTGCTAATCCTGTTA
CCAGTGGCTGCTGCCAGTGGCGATAAGTCGTGTCTTACCGGGTTGGACTCAAGACGATAGTTAC
CGGATAAGGCGCAGCGGTCGGGCTGAACGGGGGGTTCGTGCACACAGCCCAGCTTGGAGCGA
ACGACCTACACCGAACTGAGATACCTACAGCGTGAGCATTGAGAAAGCGCCACGCTTCCCGAA
GGGAGAAAGGCGGACAGGTATCCGGTAAGCGGCAGGGTCGGAACAGGAGAGCGCACGAGGG
AGCTTCCAGGGGGAAACGCCTGGTATCTTTATAGTCCTGTCGGGTTTCGCCACCTCTGACTTGA
GCGTCGATTTTTGTGATGCTCGTCAGGGGGGCGGAGCCTATGGAAAAACGCCAGCAACGCGGC
CTTTTTACGGTTCCTGGCCTTTTGCTGGCCTTTTGCTCACATGTTCTTTCCTGCGTTATCCCCTGAT
TCTGTGGATAACCGTATTACCGCCTTTGAGTGAGCTGATACCGCTCGCCGCAGCCGAACGACCG
AGCGCAGCGAGTCAGTGAGCGAGGAAGCGGAAGAGCGCCCAATACGCAAACCGCCTCTCCCC
GCGCGTTGGCCGATTCATTAATGCAGCTGGCACGACAGGTTTCCCGACTGGAAAGCGGGCAGT
GAGCGCAACGCAATTAATGTGAGTTAGCTCACTCATTAGGCACCCCAGGCTTTACACTTTATGCT
TCCGGCTCGTATGTTGTGTGGAATTGTGAGCGGATAACAATTTCACACAGGAAACAGCTATGAC
CATGATTACGAATTGTACGCGGTCGATCTTGACGGCTGGCGAGAGGTGCGGGGAGGATCTGAC
CGACGCGGTCCACACGTGGCACCGCGATGCTGTTGTGGGCACAATCGTGCCGGTTGGTAGGAT
CGACGGCCATGGGTTTTAGAGCTAGAAATAGCAAGTTAAAATAAGGCTAGTCCGTTATCAACTT
GAAAAAGTGGCACCGAGTCGGTGCTTTTTTTACGTAGGCGTCAGCTACGATGTTCCGGGGACTG
CTGATCCGGTCAGCAGGTGGAAGAGGGACTGGATTCCAAAGTTCTCAATGCTGCTTGCTGTTCT
TGAATGGGGGGTCGTTGACGACGACATGGCTCGATTGGCGCGACAAGTTGCTGCGATTCTCAC
CAATAAAAAACGCCCGGCGGCAACCGAGCGTTCTGAACAAATCCAGATGGAGTTCTGAGGTCA
TTACTGGACCGGATCGGGGATCTGGGCTGAGGGAGCCGACGGCACGCGGCGGCTCACGGCGT
GGCACGCGGAACGTCCGGGCTTGCACCTCACGTCACGTGAGGAGGCAGCGTGGACGGCGTCA
GAGAAGGGAGCGGACATATGGACAAGAAGTACTCCATCGGCCTCGACATCGGCACCAACTCC
GTGGGCTGGGCGGTCATCACCGACGAGTACAAGGTCCCCTCCAAGAAGTTCAAGGTCCTGGGC
AACACCGACCGGCACTCGATCAAGAAGAACCTGATCGGCGCCCTGCTCTTCGACAGCGGCGAG
ACCGCCGAGGCGACCCGCCTGAAGCGGACCGCGCGTCGCCGCTACACCCGGCGCAAGAACCG
CATCTGCTACCTGCAGGAAATCTTCTCCAACGAGATGGCCAAGGTGGACGACTCGTTCTTCCAC
CGCCTGGAGGAGAGCTTCCTGGTGGAGGAGGACAAGAAGCACGAGCGCCACCCGATCTTCGG
CAACATCGTGGACGAGGTGGCCTACCACGAGAAGTACCCCACCATCTACCACCTCCGCAAGAA
GCTGGTGGACTCGACCGACAAGGCGGACCTGCGGCTCATCTACCTGGCCCTCGCGCACATGAT
CAAGTTCCGCGGCCACTTCCTCATCGAGGGCGACCTGAACCCGGACAACTCCGACGTGGACAA
GCTCTTCATCCAGCTGGTGCAGACCTACAACCAGCTGTTCGAGGAGAACCCCATCAACGCCAG
CGGCGTGGACGCCAAGGCGATCCTCTCCGCGCGCCTGAGCAAGTCCCGGCGCCTGGAGAACC
TCATCGCCCAGCTGCCGGGCGAGAAGAAGAACGGCCTCTTCGGCAACCTGATCGCGCTGTCGC
TCGGCCTGACCCCCAACTTCAAGAGCAACTTCGACCTGGCCGAGGACGCGAAGCTCCAGCTGT
CCAAGGACACCTACGACGACGACCTGGACAACCTGCTCGCCCAGATCGGCGACCAGTACGCG
GACCTCTTCCTGGCCGCGAAGAACCTCTCGGACGCCATCCTGCTCAGCGACATCCTGCGGGTCA
ACACCGAGATCACCAAGGCCCCGCTGTCGGCGAGCATGATCAAGCGGTACGACGAGCACCAC
CAGGACCTGACCCTGCTCAAGGCCCTCGTGCGCCAGCAGCTGCCCGAGAAGTACAAGGAAATC

TTCTTCGACCAGTCCAAGAACGGCTACGCCGGCTACATCGACGGCGGCGCGTCGCAGGAGGAG
TTCTACAAGTTCATCAAGCCGATCCTGGAGAAGATGGACGGCACCGAGGAGCTGCTCGTCAAG
CTGAACCGCGAGGACCTGCTCCGCAAGCAGCGGACCTTCGACAACGGCTCCATCCCGCACCAG
ATCCACCTGGGCGAGCTCCACGCCATCCTCCGGCGCCAGGAGGACTTCTACCCCTTCCTGAAG
GACAACCGCGAGAAGATCGAGAAGATCCTGACCTTCCGCATCCCGTACTACGTCGGCCCCCTG
GCCCGCGGCAACTCCCGGTTCGCGTGGATGACCCGGAAGTCGGAGGAGACCATCACCCCGTG
GAACTTCGAGGAGGTCGTGGACAAGGGCGCGTCCGCGCAGTCGTTCATCGAGCGCATGACCAA
CTTCGACAAGAACCTCCCGAACGAGAAGGTCCTGCCCAAGCACTCCCTGCTCTACGAGTACTTC
ACCGTGTACAACGAGCTGACCAAGGTCAAGTACGTGACCGAGGGCATGCGGAAGCCGGCCTTC
CTGTCGGGCGAGCAGAAGAAGGCGATCGTGGACCTGCTCTTCAAGACCAACCGCAAGGTCACC
GTGAAGCAGCTGAAGGAGGACTACTTCAAGAAGATCGAGTGCTTCGACTCCGTCGAGATCAGC
GGCGTGGAGGACCGCTTCAACGCCTCCCTGGGCACCTACCACGACCTGCTCAAGATCATCAAG
GACAAGGACTTCCTCGACAACGAGGAGAACGAGGACATCCTGGAGGACATCGTCCTCACCCTG
ACCCTCTTCGAGGACCGCGAGATGATCGAGGAGCGGCTCAAGACCTACGCCCACCTGTTCGAC
GACAAGGTGATGAAGCAGCTGAAGCGTCGCCGCTACACCGGCTGGGGCCGCCTCTCCCGGAA
GCTGATCAACGGCATCCGGGACAAGCAGAGCGGCAAGACCATCCTGGACTTCCTCAAGTCCGA
CGGCTTCGCCAACCGCAACTTCATGCAGCTCATCCACGACGACAGCCTGACCTTCAAGGAGGA
CATCCAGAAGGCCCAGGTCTCGGGCCAGGGCGACAGCCTCCACGAGCACATCGCCAACCTGG
CGGGCTCCCCGGCGATCAAGAAGGGCATCCTCCAGACCGTCAAGGTCGTGGACGAGCTGGTCA
AGGTGATGGGCCGCCACAAGCCCGAGAACATCGTGATCGAGATGGCCCGGGAGAACCAGACC
ACCCAGAAGGGCCAGAAGAACTCGCGCGAGCGGATGAAGCGGATCGAGGAGGGCATCAAGG
AGCTCGGCAGCCAGATCCTGAAGGAGCACCCGGTCGAGAACACCCAGCTGCAGAACGAGAAG
CTGTACCTCTACTACCTGCAGAACGGCCGCGACATGTACGTGGACCAGGAGCTCGACATCAAC
CGGCTGTCCGACTACGACGTGGACCACATCGTGCCGCAGTCCTTCCTGAAGGACGACTCGATC
GACAACAAGGTCCTGACCCGCTCGGACAAGAACCGGGGCAAGTCCGACAACGTGCCCTCGGA
GGAGGTCGTGAAGAAGATGAAGAACTACTGGCGCCAGCTGCTCAACGCCAAGCTCATCACCCA
GCGCAAGTTCGACAACCTGACCAAGGCCGAGCGGGGCGGCCTGAGCGAGCTCGACAAGGCGG
GCTTCATCAAGCGCCAGCTGGTCGAGACCCGGCAGATCACCAAGCACGTGGCCCAGATCCTGG
ACTCCCGGATGAACACCAAGTACGACGAGAACGACAAGCTGATCCGCGAGGTCAAGGTGATCA
CCCTCAAGAGCAAGCTGGTCTCCGACTTCCGCAAGGACTTCCAGTTCTACAAGGTCCGGGAGAT
CAACAACTACCACCACGCCCACGACGCGTACCTGAACGCCGTCGTGGGCACCGCGCTGATCAA
GAAGTACCCGAAGCTGGAGTCCGAGTTCGTCTACGGCGACTACAAGGTCTACGACGTGCGCAA
GATGATCGCCAAGAGCGAGCAGGAGATCGGCAAGGCCACCGCGAAGTACTTCTTCTACTCCAA
CATCATGAACTTCTTCAAGACCGAGATCACCCTGGCCAACGGCGAGATCCGCAAGCGGCCCCT
GATCGAGACCAACGGCGAGACCGGCGAGATCGTCTGGGACAAGGGCCGCGACTTCGCCACCG
TCCGGAAGGTGCTGTCGATGCCGCAGGTCAACATCGTGAAGAAGACCGAGGTGCAGACCGGC
GGCTTCAGCAAGGAGTCCATCCTCCCCAAGCGCAACAGCGACAAGCTGATCGCCCGGAAGAA
GGACTGGGACCCGAAGAAGTACGGCGGCTTCGACAGCCCCACCGTCGCCTACTCCGTGCTGGT
CGTGGCGAAGGTCGAGAAGGGCAAGAGCAAGAAGCTGAAGTCCGTGAAGGAGCTGCTCGGCA
TCACCATCATGGAGCGCTCCTCGTTCGAGAAGAACCCGATCGACTTCCTGGAGGCCAAGGGCT
ACAAGGAGGTCAAGAAGGACCTCATCATCAAGCTGCCCAAGTACAGCCTGTTCGAGCTGGAGA
ACGGCCGCAAGCGGATGCTCGCCTCCGCGGGCGAGCTGCAGAAGGGCAACGAGCTGGCCCTC
CCGTCGAAGTACGTCAACTTCCTGTACCTCGCGTCCCACTACGAGAAGCTGAAGGGCTCGCCC
GAGGACAACGAGCAGAAGCAGCTCTTCGTGGAGCAGCACAAGCACTACCTGGACGAGATCAT

CGAGCAGATCAGCGAGTTCAGCAAGCGCGTCATCCTGGCCGACGCGAACCTCGACAAGGTGCT
GTCCGCCTACAACAAGCACCGCGACAAGCCGATCCGGGAGCAGGCGGAGAACATCATCCACC
TGTTCACCCTCACCAACCTGGGCGCCCCCGCCGCGTTCAAGTACTTCGACACCACCATCGACCG
CAAGCGGTACACCTCCACCAAGGAGGTCCTCGACGCGACCCTGATCCACCAGAGCATCACCGG
CCTGTACGAGACCCGCATCGACCTGTCCCAGCTCGGCGGCGACTGAAAGCTTTCTAGAGGATC
CCCGGGTACCGAGCTCGAATTCCCCAGATCTAAAGTTTTGTCGTCTTTCCAGACGTTAGTAAATG
AATTTTCTGTATGAGGTTTTGCTAAACAACTTTCAACAGTTTCAGCGGAGTGAGAATAGAAAGGA
ACAACTAAAGGAATTGCGAATAATAATTTTTTCACGTTGAAAATCTCCAAAAAAAAAGGCTCCA
AAAGGAGCCTTTAATTGTATCGGTTTATCAGCTTGCTTTCGAGGTGAATTTCTTAAACAGCTTGAT
ACCGATAGTTGCGCCGACAATGACAACAACCATCGCCCACGCATAACCGATATATTCGGTCGCT
GAGGCTTGCAGGGAGTCAAAGGCCGCTTTTGCGGGATCTCGTCGAAGGCACTAGAAGGCCTAT
ATGCGGGGATCGACCGCGCGGGTCCCGGACGGGGAAGAGCGGGGAGCTTTGCCAGAGAGCG
ACGACTTCCCCTTGCGTTGGTGATTGCCGGTCAGGGCAGCCATCCGCCATCGTCGCGTAGGGTG
TCACACCCCAGGAATCGCGTCACTGAACACAGCAGCCGGTAGGACGACCATGACTGAGTTGGA
CACCATCGCAAATCCGTCCGATCCCGCGGTGCAGCGGATCATCGATGTCACCAAGCCGTCGCG
ATCCAACATAAAGACAACGTTGATCGAGGACGTCGAGCCCCTCATGCACAGCATCGCGGCCGG
GGTGGAGTTCATCGAGGTCTACGGCAGCGACAGCAGTCCTTTTCCATCTGAGTTGCTGGATCTG
TGCGGGCGGCAGAACATACCGGTCCGCCTCATCGACTCCTCGATCGTCAACCAGTTGTTCAAGG
GGGAGCGGAAGGCCAAGACATTCGGCATCGCCCGCGTCCCTCGCCCGGCCAGGTTCGGCGAT
ATCGCGAGCCGGCGTGGGGACGTCGTCGTTCTCGACGGGGTGAAGATCGTCGGGAACATCGG
CGCGATAGTACGCACGTCGCTCGCGCTCGGAGCGTCGGGGATCATCCTGGTCGACAGTGACAT
CACCAGCATCGCGGACCGGCGTCTCCAAAGGGCCAGCCGAGGTTACGTCTTCTCCCTTCCCGTC
GTTCTCTCCGGTCGCGAGGAGGCCATCGCCTTCATTCGGGACAGCGGTATGCAGCTGATGACG
CTCAAGGCGGATGGCGACATTTCCGTGAAGGAACTCGGGGACAATCCGGATCGGCTGGCCTTG
CTGTTCGGCAGCGAAAAGGGTGGGCCTTCCGACCTGTTCGAGGAGGCGTCTTCCGCCTCGGTTT
CCATCCCCATGATGAGCCAGACCGAGTCTCTCAACGTTTCCGTTTCCCTCGGAATCGCGCTGCA
CGAGAGGATCGACAGGAATCTCGCGGCCAACCGATAAGCGCCTCTGTTCCTCGGACGCTCGGT
TCCTCGACCTCGATTCGTCAGTGATGATCAACAGGACCCAGAAATGGCACGAGCCCGGGATTG
GCCTCCCGGGCTCGCCGCCCCCGAACCGGCGCAGCATGCCGAGTGAGGACGTTTGCTCTGTGA
CGAGCGTACGAGAGAACAGCGCGTCGACACCACCGCGCCCCGCCGGACCGAAGTGCCACTGC
GGCACCCCGATCGTCCACATCCCCGGGAAGCGCCCCAAAATCTACTGCTCGGACGCCTGCCGA
AAGCGCGCAAAGCGTCTCATTGCGGACCAGTCACGCAGCGTAGCAAGCACGACTACAGCGGG
TAATCCGGACGCGGGCTTTAGTAGTAAGGGAAAATCTCCGTCCACTGCAGGTCAGAGCGCTGT
AACTGGAACGGGTTCTACCCCAAACGAGGGTGCCGCGACAGCCGGGCGGGAGGACCCGCGCG
ACGAGCGGTTCGCCCGGCGGGACCGGCACCAGACGGTGTCGCTGAACGAGGCGTTCAAGGGC
TGCGGGACCCGGCTCACTGCCGGGACGGCCGAGCTGATGTGGAAGCCGGGCGAGGCGACGTG
GGGCAACACGTGCCGCTGCAACAACGTCCACACCTGCCCGTGGTGCATGAGCCGCATCCTGGC
CGTACGGGGCTCGAACGTGCAGCTCGCCGCCGACGGTCTCGCGGACGCCGGGTACGGGCTGC
ACCTTGGCACGAACACGTTGCGCCACTTCGAGCGCATGGCGTTCGGCACCGTCCGCAAGGGAA
TGCGGCACGGCCTGGTCGCGGTCTTGCACGACGGCTGGAAGGGCGCGTACGGCTCGTCCGGC
CGCCGCTGGCGCACGATGCGCGACGACTTCGGGATCATCGGCTACGAGCGGGCCTTCGAGGA
CACCTTCGGCTGGGGCTCCGGCTGGCACCTGCACTGGCACACGCTCTGGGTCACCCGCGAGGT
GCTCGGCCCGGACGCCCAGGCCGCGTTCCGCGACGCACTCGCCGGAGCCTGGGCCGCCGGGG
TCGAATCCGCGGGCGGCTACACCGTCAGCGAGACGTGCGACCGGCCCGGTTGCTCCTGTGAGG

GCAAGGGCCACGGCACCGACGTGCGCCCACTCAACGGCGCGGACGCCGCTGACGGCGATGCG
GGCAAGCAGGCCCGCTACCTCTACAAGGACGGCGACAAGACCAAGGGCGGCGTCGCCAAGAT
CGGCCTGGAGCTGGCCGGACAGAACTTCAAGGCCGGTCGCGGCGACGACCGCATGGGACCGC
TCGACCTGGGCGACGCGGCGGCCGCCGAGCTGCAGCGGCTTCGTCGGCCCGGCCCGTTCGTC
GAGAAGTACCGCGAGCGCGAGTTCGGCGTCTTCCAGGTCCGCAAGCACTACCGGTCGCAGAAC
CTGAACCGGCTGATCAAGGAACTCGGCATCCAGCAGGACGTCCGCACCGAGGAGGAGATCAC
CGACGACACCGAGGGCCTGGTCGCCATCGCGGTCATCCCGGCCTACATCTGGTACCGATACAT
CGCCCGCGTCGCCGGACGTCGCCTCGACCTCATCAAGGTCGCCGAGACGTACGGCCTACCGGG
CGTCCGGCGGCTCGTCGAGTCCTGGGGCCTGGTGTGGGGCAAGGACGTCCTGGACCCGCCCG
CCCCGGAAGCGCCGGCCGCGCCGGGCGACCTTGACGCTGACCAGATGAGGTTCGAGGTCATG
TCCGAGGAGGAAGCTGCTTTCCGCGAGGCGCGCCGGAAGGCCAACGAGGCCCGCACGGAGG
AGCTGGCCGCTTCGCTCGACCGAGTGCGTCAGCCGAAGAAGGAGGCGATCCGCCCGACGATC
AGCCTTCGGAAGCGCCTGAAGCCGAAGCCGGTCACCGTCGACGTCAAGACTCCCCCGCCCGG
CGCCGCGTCGCCCGTGTGCCGCCGCTGCAAGGGCAAGCTCGCCCCGGTACTCCAGCCCTGGG
GCCGGCTCCCCGGAGACTGCCTCCGCGTCGACACAGCGGTCGCGTAGGGCCGCTGAGGGACC
GCCATGAGACCGCCACCCCGGACATCCGGGTGGCGGTCTCATGGCGGTCCCGGCGCGAAAGC
CGAGGGTCTGCCTGCCGTGAGGTGGCCGGCGGACCTGGGTTACTGCTGGAATCCCGCGCCCAG
TCCGAGCGTCCGCCGGCGCTTTTCACCTGGCTCCGGGTGCTCGTCGATGAGCACCA

PUN-3FLAG

agattacgggggaattccttttcccaccgctcattcacatttcccattcatcggcccgccgtaaataaaatagacaccccctcaaa
caccctctttcccccaacctcgtgttgttcggaggcgcacaacacacacaaaccagatctcccccaaaatccacccgtcggcacct
ccgcttcaaggtacgccgctcgtcctcccccccccccctctctaccttctctagatcggcgttccggtccatggttagggcccggt
agttctacttctgttcatgtttgtgttagatccgtgtttgtgttagatccgtgctgctagcgttcgtacacggatgcgacctgtacgtc
agacacgttctgattgctaacttgccagtgtttctctttggggaatcctgggatggctctagccgttccgcagacgggatcgatttc
atgattttttttgtttcgttgcatagggtttggtttgcccttttcctttatttcaatatatgccgtgcacttgtttgtcgggtcatcttttcatg
cttttttttgtcttggttgtgatgatgtggtctggttgggcggtcgttctagatcggagtagaattctgtttcaaactacctggtggatt
tattaattttggatctgtatgtgtgtgccatacatattcatagttacgaattgaagatgatggatggaaatatcgatctaggataggt
atacatgttgatgcgggttttactgatgcatatacagagatgctttttgttcgcttggttgtgatgatgtggtgtggttgggcggtc
gttcattcgttctagatcggagtagaatactgtttcaaactacctggtgtatttattaattttggaactgtatgtgtgtgtcatacatctt
catagttacgagtttaagatggatggaaatatcgatctaggataggtatacatgttgatgtgggttttactgatgcatatacatgat
ggcatatgcagcatctattcatatgctctaaccttgagtacctatctattataataaacaagtatgttttataattattttgatcttgatat
acttggatgatggcatatgcagcagctatatgtggatttttttagccctgccttcatacgctatttatttgcttggtactgtttcttttgtc
gatgctcaccctgttgtttggtgttacttctgcaggtcgactctagaggatccatggattacaaggatcatgatggagactacaag
gatcatgatattgattacaaagatgatgacgataagggatccccgggtaccgagctcgaatttccccgatcgttcaaacatttgg
caataaagtttcttaagattgaatcctgttgccggtcttgcgatgattatcatataatttctgttgaattacgttaagcatgtaataatta
acatgtaatgcatgacgttatttatgagatgggtttttatgattagagtcccgcaattatacatttaatacgcgatagaaaacaaaat
atagcgcgcaaactaggataaattatcgcgcgcggtgtcatctatgttactagatcgggaattcgtaatcatgtcatagctgtttcc
tgtgtgaaattgttatccgctcacaattccacacaacatacgagccggaagcataaagtgtaaagcctggggtgcctaatgagt
gagctaactcacattaattgcgttgcgctcactgcccgctttccagtcgggaaacctgtcgtgccagctgcattaatgaatcggcc
aacgcgcggggagaggcggtttgcgtattggctagagcagcttgccaacatggtggagcacgacactctcgtctactccaag
aatatcaaagatacagtctcagaagaccaaagggctattgagacttttcaacaaagggtaatatcgggaaacctcctcggattcc
attgcccagctatctgtcacttcatcaaaaggacagtagaaaaggaaggtggcacctacaaatgccatcattgcgataaaggaa
aggctatcgttcaagatgcctctgccgacagtggtcccaaagatggacccccacccacgaggagcatcgtggaaaaagaaga

cgttccaaccacgtcttcaaagcaagtggattgatggggaacatggggggagcacgacactctcgtctactccaagaatatcaa
agatacagtctcagaagaccaaagggctattgagacttttcaacaaagggtaatatcgggaaacctcctcggattccattgccca
gctatctgtcacttcatcaaaaggacagtagaaaaggaaggtggcacctacaaatgccatcattgcgataaaggaaaggctatc
gttcaagatgcctctgccgacagtggtcccaaagatggacccccacccacgaggagcatcgtggaaaaagaagacgttccaa
ccacgtcttcaaagcaagtggattgatgtgatatctccactgacgtaagggatgacgcacaatcccactatccttcgcaagaccct
tcctctatataaggaagttcatttcatttggagaggacacgctgaaatcaccagtctctctctacaaatctatctctctcgagctttcg
cagatccgggggcaatgagatatgaaaaagcctgaactcaccgcgacgtctgtcgagaagtttctgatcgaaaagttcgaca
gcgtctccgacctgatgcagctctcggagggcgaagaatctcgtgctttcagcttcgatgtaggagggcgtggatatgtcctgc
gggtaaatagctgcgccgatggtttctacaaagatcgttatgtttatcggcactttgcatcggccgcgctcccgattccggaagtg
cttgacattggggagtttagcgagagcctgacctattgcatctcccgccgttcacagggtgtcacgttgcaagacctgcctgaaa
ccgaactgcccgctgttctacaaccggtcgcggaggctatggatgcgatcgctgcggccgatcttagccagacgagcgggttc
ggcccattcggaccgcaaggaatcggtcaatacactacatggcgtgatttcatatgcgcgattgctgatccccatgtgtatcactg
gcaaactgtgatggacgacaccgtcagtgcgtccgtcgcgcaggctctcgatgagctgatgctttgggccgaggactgccccg
aagtccggcacctcgtgcacgcggatttcggctccaacaatgtcctgacggacaatggccgcataacagcggtcattgactgg
agcgaggcgatgttcggggattcccaatacgaggtcgccaacatcttcttctggaggccgtggttggcttgtatggagcagca
gacgcgctacttcgagcggaggcatccggagcttgcaggatcgccacgactccgggcgtatatgctccgcattggtcttgacc
aactctatcagagcttggttgacggcaatttcgatgatgcagcttgggcgcagggtcgatgcgacgcaatcgtccgatccgga
gccgggactgtcgggcgtacacaaatcgcccgcagaagcgcggccgtctggaccgatggctgtgtagaagtactcgccgat
agtggaaaccgacgccccagcactcgtccgagggcaaagaaatagagtagatgccgaccgggatctgtcgatcgacaagct
cgagtttctccataataatgtgtgagtagttcccagataagggaattagggttcctatagggtttcgctcatgtgttgagcatataa
gaaacccttagtatgtatttgtatttgtaaaatacttctatcaataaaatttctaattcctaaaaccaaaatccagtactaaaatccaga
tcccccgaattaattcggcgttaattcagtacattaaaaacgtccgcaatgtgttattaagttgtctaagcgtcaatttgtttacacca
caatatatcctgccaccagccagccaacagctccccgaccggcagctcggcacaaaatcaccactcgatacaggcagcccatc
agtccgggacggcgtcagcgggagagccgttgtaaggcggcagactttgctcatgttaccgatgctattcggaagaacggca
actaagctgccgggtttgaaacacggatgatctcgcggagggtagcatgttgattgtaacgatgacagagcgttgctgcctgtg
atcaccgcggtttcaaaatcggctccgtcgatactatgttatacgccaactttgaaaacaactttgaaaaagctgttttctggtattta
aggttttagaatgcaaggaacagtgaattggagttcgtcttgttataattagcttcttggggtatctttaaatactgtagaaaagag
gaaggaaataataaatggctaaaatgagaatatcaccggaattgaaaaaactgatcgaaaaataccgctgcgtaaaagatacg
gaaggaatgtctcctgctaaggtatataagctggtgggagaaaatgaaaacctatatttaaaaatgacggacagccggtataa
agggaccacctatgatgtggaacgggaaaaggacatgatgctatggctggaaggaaagctgcctgttccaaaggtcctgcac
tttgaacggcatgatggctggagcaatctgctcatgagtgaggccgatggcgtcctttgctcggaagagtatgaagatgaaca
aagccctgaaaagattatcgagctgtatgcggagtgcatcaggctctttcactccatcgacatatcggattgtccctatacgaata
gcttagacagccgcttagccgaattggattacttactgaataacgatctggccgatgtggattgcgaaaactgggaagaagaca
ctccatttaaagatccgcgcgagctgtatgatttttttaaagacggaaaagcccgaagaggaacttgtcttttcccacggcgacctg
ggagacagcaacatctttgtgaaagatggcaaagtaagtggctttattgatcttgggagaagcggcagggcggacaagtggt
atgacattgccttctgcgtccggtcgatcagggaggatatcggggaagaacagtatgtcgagctatttttttgacttactggggat
caagcctgattgggagaaaataaaatattatattttactggatgaattgttttagtacctagaatgcatgaccaaaatcccttaacg
ctgagagatcccctcataatttccccaaagcgtaaccatgtgtgaataaattttgagctagtagggttgcagccacgagtaagtct
tcccttgttattgtgtagccagaatgccgcaaaacttccatgcctaagcgaactgttgagagtacgtttcgatttctgactgtgttag
cctggaagtgcttgtcccaaccttgtttctgagcatgaacgcccgcaagccaacatgttagttgaagcatcagggcgattagcag
catgatatcaaaacgctctgagctgctcgttcggctatggcgtaggcctagtccgtaggcaggacttttcaagtctcggaaggttt
cttcaatctgcattcgcttcgaatagatattaacaagttgtttgggtgttcgaatttcaacaggtaagttagttgctagaatccatgg
ctcctttgccgacgctgagtagattttaggtgacgggtggtgacaatgagtccgtgtcgagcgctgatttttcggcctttagagc
gagatttatacaatagaatttggcatgagattggattgcttttagtcagcctcttatagcctaaagtctttgagtgactagatgacat

atcatgtaagttgctgataggtttccagttttccgctcctaggtctgcatattgtacttttcctcttactcgacttaaccagtaccaaccc
agcttctcaacggatttataccatggcactttaaagccagcatcactgacaatgagcggtgtggtgttactcggtagaatgctcgc
aaggtcggctagaaattggtcatgagctttctttgaacattgctctgaaagcgggaacgctttctcataaagagtaacagaacga
ccgtgtagtgcgactgaagctcgcaataccataagccgtttttgctcacggatatcagaccagtcaacaagtacaatgggcatcg
tattgcccgaacagataaagctagcatgccaacggtatacagcgagtcgctctttgtggaggtgacgattacctaacaatcggtc
gattcgtttgatgttatgttttgttctcgctttggttggcaggttacggccaagttcggtaagagtgagagttttacagtcaagtaag
gcgtggcaagccaacgttaagctgttgagtcgttttaagtgtaattcggggcagaattggtaaagagagtcgtgtaaaatatcg
agttcgcacattttgttgtctgattattgatttttggcgaaaccatttgatcatatgacaagatgtgtatctaccttaacttaatgattttg
ataaaaatcattaggggattcatcagcccttaacgtgagttttcgttccactgagcgtcagaccccgtagaaaagatcaaaggat
cttcttgagatcctttttttctgcgcgtaatctgctgcttgcaaacaaaaaaaccaccgctaccagcggtggtttgtttgccggatca
agagctaccaactctttttccgaaggtaactggcttcagcagagcgcagataccaaatactgtccttctagtgtagccgtagttag
gccaccacttcaagaactctgtagcaccgcctacatacctcgctctgctaatcctgttaccagtggctgctgccagtggcgataag
tcgtgtcttaccgggttggactcaagacgatagttaccggataaggcgcagcggtcgggctgaacggggggttcgtgcacac
agcccagcttggagcgaacgacctacaccgaactgagatacctacagcgtgagctatgagaaagcgccacgcttcccgaagg
gagaaaggcggacaggtatccggtaagcggcagggtcggaacaggagagcgcacgagggagcttccagggggaaacg
cctggtatctttatagtcctgtcgggtttcgccacctctgacttgagcgtcgatttttgtgatgctcgtcaggggggcggagcctat
ggaaaaacgccagcaacgcggcctttttacggttcctggccttttgctggccttttgctcacatgttctttcctgcgttatcccctgatt
ctgtggataaccgtattaccgcctttgagtgagctgataccgctcgccgcagccgaacgaccgagcgcagcgagtcagtgagc
gaggaagcggaagagcgcctgatgcggtattttctccttacgctctgtgcggtatttcacaccgcatatggtgcactctcagtaca
atctgctctgatgccgcatagttaagccagtatacactccgctatcgctacgtgactgggtcatggctgcgccccgacacccgcc
aacacccgctgacgcgccctgacgggcttgtctgctcccggcatccgcttacagacaagctgtgaccgtctccgggagctgcat
gtgtcagaggttttcaccgtcatcaccgaaacgcgcgaggcagggtgccttgatgtgggcgccggcggtcgagtggcgacg
gcgcggcttgtccgcgccctggtagattgcctggccgtaggccagccatttttgagcggccagcggccgcgataggccgacg
cgaagcggcggggcgtagggagcgcagcgaccgaagggtaggcgctttttgcagctcttcggctgtgcgctggccagaca
gttatgcacaggccaggcgggttttaagagttttaataagttttaaagagttttaggcggaaaaatcgcctttttctcttttatatca
gtcacttacatgtgtgaccggttcccaatgtacggctttgggttcccaatgtacgggttccggttcccaatgtacggctttgggttc
ccaatgtacgtgctatccacaggaaagagaccttttcgacctttttcccctgctagggcaatttgccctagcatctgctccgtacatt
aggaaccggcggatgcttcgccctcgatcaggttgcggtagcgcatgactaggatcgggccagcctgccccgcctcctccttca
aatcgtactccggcaggtcatttgacccgatcagcttgcgcacggtgaaacagaacttcttgaactctccggcgctgccactgcg
ttcgtagatcgtcttgaacaaccatctggcttctgccttgcctgcggcgcggcgtgccaggcggtagagaaaacggccgatgcc
gggatcgatcaaaaagtaatcggggtgaaccgtcagcacgtccgggttcttgccttctgtgatctcgcggtacatccaatcagct
agctcgatctcgatgtactccggccgcccggtttcgctctttacgatcttgtagcggctaatcaaggcttcaccctcggataccgtc
accaggcggccgttcttggccttcttcgtacgctgcatggcaacgtgcgtggtgtttaaccgaatgcaggtttctaccaggtcgtc
tttctgctttccgccatcggctcgccggcagaacttgagtacgtccgcaacgtgtggacggaacacgcggccgggcttgtctccc
ttcccttcccggtatcggttcatggattcggttagatgggaaaccgccatcagtaccaggtcgtaatcccacacactggccatgcc
ggccggccctgcggaaacctctacgtgcccgtctggaagctcgtagcggatcacctcgccagctcgtcggtcacgcttcgaca
gacggaaaacggccacgtccatgatgctgcgactatcgcgggtgcccacgtcatagagcatcggaacgaaaaaatctggttg
ctcgtcgcccttgggcggcttcctaatcgacggcgcaccggctgccggcggttgccgggattctttgcggattcgatcagcggc
cgcttgccacgattcaccggggcgtgcttctgcctcgatgcgttgccgctgggcggcctgcgcggccttcaacttctccaccagg
tcatcacccagcgccgcgccgatttgtaccgggccggatggtttgcgaccgctcacgccgattcctcgggcttgggggttccag
tgccattgcagggccggcaggcaacccagccgcttacgcctggccaaccgcccgttcctccacacatggggcattccacggcg
tcggtgcctggttgttcttgattttccatgccgcctcctttagccgctaaaattcatctactcatttattcatttgctcatttactctggtag
ctgcgcgatgtattcagatagcagctcggtaatggtcttgccttggcgtaccgcgtacatcttcagcttggtgtgatcctccgccg
gcaactgaaagttgacccgcttcatggctggcgtgtctgccaggctggccaacgttgcagccttgctgctgcgtgcgctcggac

ggccggcacttagcgtgtttgtgcttttgctcattttctctttacctcattaactcaaatgagttttgatttaatttcagcggccagcgcc
tggacctcgcgggcagcgtcgccctcgggttctgattcaagaacggttgtgccggcggcggcagtgcctgggtagctcacgc
gctgcgtgatacgggactcaagaatgggcagctcgtacccggccagcgcctcggcaacctcaccgccgatgcgcgtgcctttg
atcgcccgcgacacgacaaaggccgcttgtagccttccatccgtgacctcaatgcgctgcttaaccagctccaccaggtcggcg
gtggcccatatgtcgtaagggcttggctgcaccggaatcagcacgaagtcggctgccttgatcgcggacacagccaagtccgc
cgcctggggcgctccgtcgatcactacgaagtcgcgccggccgatggccttcacgtcgcggtcaatcgtcgggcggtcgatg
ccgacaacggttagcggttgatcttcccgcacggccgcccaatcgcgggcactgccctggggatcggaatcgactaacagaa
catcggccccggcgagttgcagggcgcgggctagatgggttgcgatggtcgtcttgcctgacccgcctttctggttaagtacag
cgataaccttcatgcgttccccttgcgtatttgtttatttactcatcgcatcatatacgcagcgaccgcatgacgcaagctgttttactc
aaatacacatcaccttttttagacggcggcgctcggtttcttcagcggccaagctggccggccaggccgccagcttggcatcaga
caaaccggccaggatttcatgcagccgcacggttgagacgtgcgcgggcggctcgaacacgtacccggccgcgatcatctcc
gcctcgatctcttcggtaatgaaaaacggttcgtcctggccgtcctggtgcggtttcatgcttgttcctcttggcgttcattctcggc
ggccgccagggcgtcggcctcggtcaatgcgtcctcacggaaggcaccgcgccgcctggcctcggtgggcgtcacttcctcg
ctgcgctcaagtgcgcggtacagggtcgagcgatgcacgccaagcagtgcagccgcctctttcacggtgcggccttcctggtc
gatcagctcgcgggcgtgcgcgatctgtgccggggtgagggtagggcgggggccaaacttcacgcctcgggccttggcgg
cctcgcgcccgctccgggtgcggtcgatgattagggaacgctcgaactcggcaatgccggcgaacacggtcaacaccatgcg
gccggccggcgtggtggtgtcggcccacggctctgccaggctacgcaggcccgcgccggcctcctggatgcgctcggcaat
gtccagtaggtcgcgggtgctgcgggccaggcggtctagcctggtcactgtcacaacgtcgccagggcgtaggtggtcaag
catcctggccagctccgggcggtcgcgcctggtgccggtgatcttctcggaaaacagcttggtgcagccggccgcgtgcagtt
cggcccgttggttggtcaagtcctggtcgtcggtgctgacgcgggcatagcccagcaggccagcggcggcgctcttgttcatg
gcgtaatgtctccggttctagtcgcaagtattctactttatgcgactaaaacacgcgacaagaaaacgccaggaaaagggcag
ggcggcagcctgtcgcgtaacttaggacttgtgcgacatgtcgttttcagaagacggctgcactgaacgtcagaagccgactg
cactatagcagcggaggggttggatcaaagtactttgatcccgaggggaaccctgtggttggcatgcacatacaaatggacga
acggataaaccttttcacgcccttttaaatatccgattattctaataaacgctcttttctcttaggtttacccgccaatatatcctgtcaaa
cactgatagtttaattcccgatctagtaacatagatgacaccgcgcgcgataatttatcctagtttgcgcgctatattttgttttctatc
gcgtattaaatgtataattgcgggactctaatcataaaaacccatctcataaataacgtcatgcattacatgttaattattacatgctt
aacgtaattcaacagaaattatatgataatcatcgcaagaccggcaacaggattcaatcttaagaaactttattgccaaatgtttga
acgatcggggaaattcgagctggtcacctgtaattcacacgtggtggtggtggtggctagcttgtttgcctccctgctgcgg
tttttcaccgaagttcatgccagtccagcgtttttgcagcagaaaagccgccgacttcggtttgcggtcgcgagtgaagatcccttt
cttgttaccgccaacgcgcaatatgccttgcgaggtcgcaaaatcggcgaaattccatacctgttcaccgacgacggcgctgac
gcgatcaaagacgcggtgatacatatccagccatgcacactgatactcttcactccacatgtcggtgtacattgagtgcagcccg
gctaacgtatccacgccgtattcggtgatgataatcggctgatgcagtttctcctgccaggccagaagttctttttccagtaccttct
ctgccgtttccaaatcgccgctttggacataccatccgtaataacggttcaggcacagcacatcaaagagatcgctgatggtatc
ggtgtgagcgtcgcagaacattacattgacgcaggtgatcggacgcgtcgggtcgagtttacgcgttgcttccgccagtggcg
cgaaatattcccgtgcaccttgcggacgggtatccggttcgttggcaatactccacatcaccacgcttgggtgggttttgtcacgc
gctatcagctctttaatcgcctgtaagtgcgcttgctgagtttccccgttgactgcctcttcgct
